# Supplementary material for: A replication competent Plasmodium falciparum parasite completely attenuated by dual gene deletion
Source: EMBO Mol Med. 2024 Mar 21;16(4):10. doi: 10.1038/s44321-024-00057-7 (PMC11018819; doi:10.1038/s44321-024-00057-7)
Supplement: Supplementary file 1 — Appendix [file 44321_2024_57_MOESM1_ESM.pdf]

Appendix Figures and Tables

**Table of contents**	Page no.
**Appendix Figure S1:** PyLARC2 generation and analysis of mosquito stage development.	1
**Appendix Figure S2:** Gene deletion of Pf *Mei2* and Pf *LINUP* in PfLARC2 was confirmed by whole genome sequencing.	2
**Appendix Table S1:** Oligonucleotide primers used in the study.	3 – 4
**Appendix Table S2:** PfSPZ-LARC2 liver stages do not transition to productive blood stage infection after sporozoite infection in FRG huHep mice.	5

Appendix Figure S1

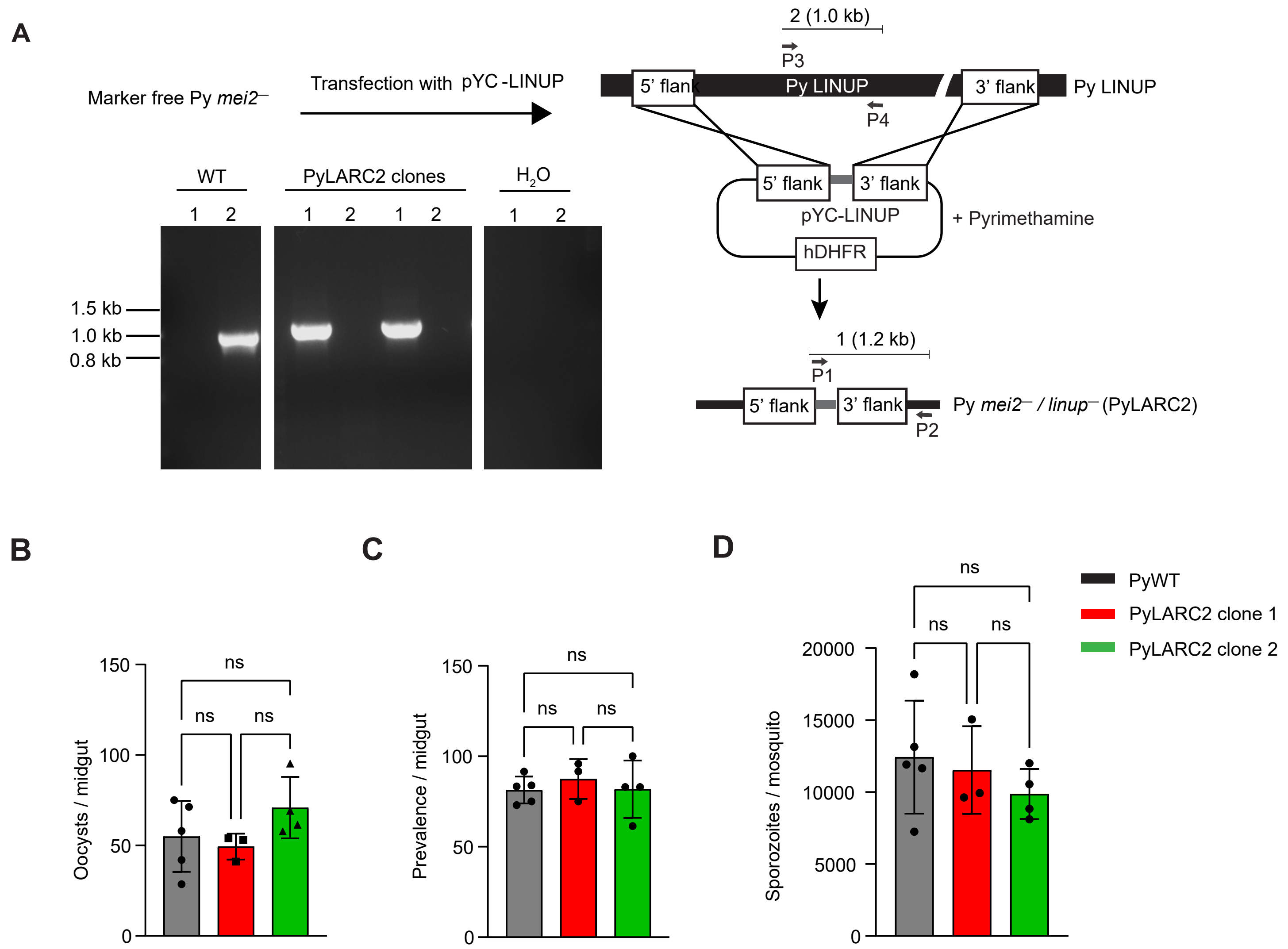

Appendix Figure S1: PyLARC2 generation and analysis of mosquito stage development. A. The schematic depicts the generation of the PyLARC2 parasite using CRISPR/Cas9-mediated gene editing. Blood stage schizonts of marker free Py mei2– were transfected with pYC-LINUP plasmid, followed by positive selection with pyrimethamine to select for Py mei2–/linup– (PyLARC2) parasites. Clonal populations were derived by limiting dilution cloning. Agarose gel electrophoresis shows the PCR products corresponding to the gene deletion of Py LINUP in clones 1 and 2 of PyLARC2. Primers used to verify the gene deletion are indicated and the sizes of the PCR amplicons are shown in kilobases. B. PyLARC2 clones 1 and 2 did not have defects in mosquito infectivity as counts for B., oocysts/midgut, C., oocyst prevalence and D., salivary gland sporozoites/mosquito were comparable between PyLARC2 clones 1 (red) and 2 (green) to PyWT (black). Data is represented as mean +/- SD, for at least n = 3 biological replicates. Statistical analysis was carried out using one way ANOVA using Tukey's multiple comparison test. P>0.05 is taken as not significant.

Appendix Figure S2

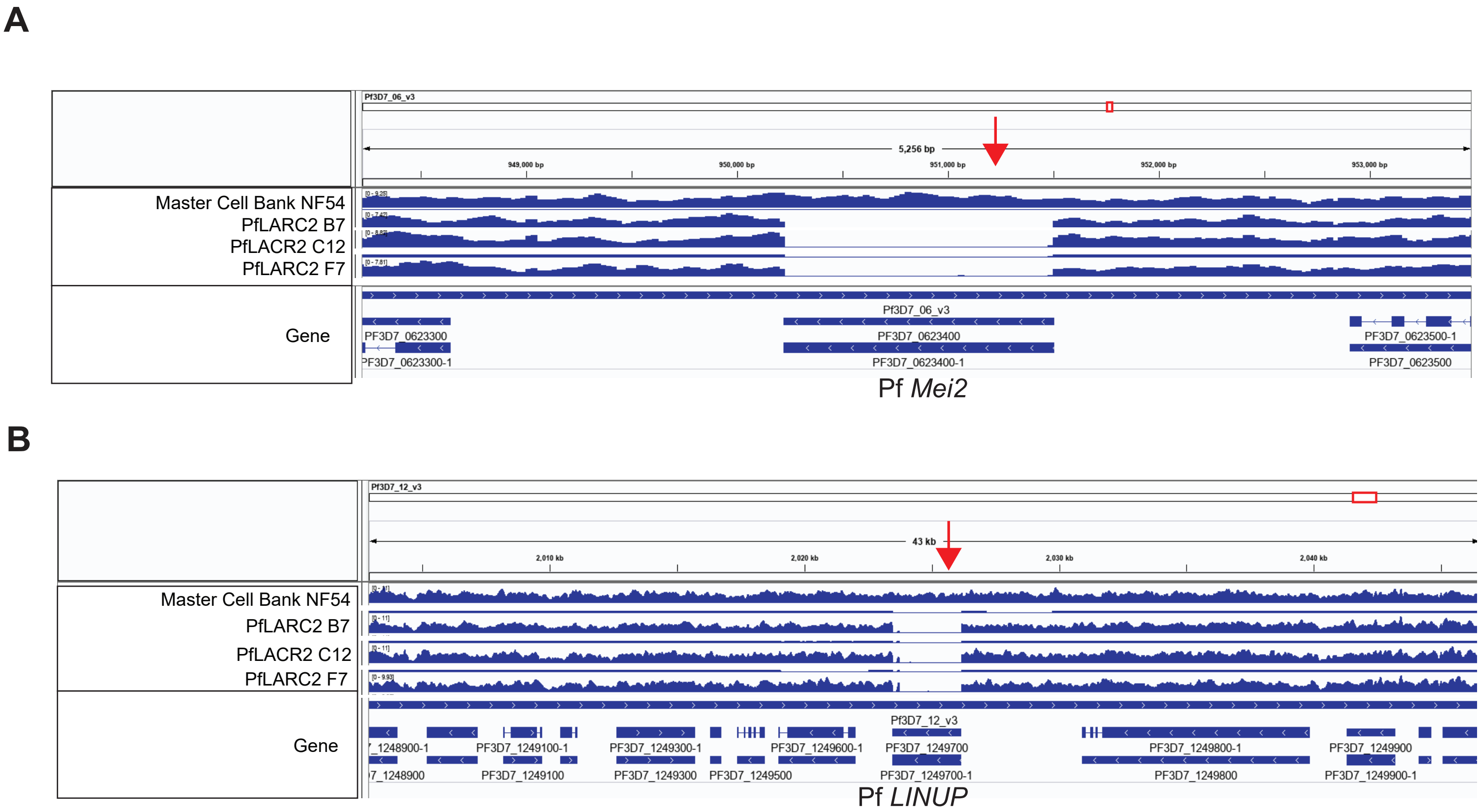

Appendix Figure S2: Gene deletion of Pf Mei2 and Pf LINUP in PfLARC2 was confirmed by whole genome sequencing. A. The absence of Pf Mei2 (PF3D7_0623400) in chromosome 6 and B. Pf LINUP (PF3D7_1249700) in chromosome 12 in PfLARC2 clones F7, C12 and B7 was confirmed by whole genome sequencing when compared to PfNF54-WT.

Appendix Table S1: Oligonucleotide primers used in the study.

**Primers for Py LARC2 generation and genotyping**	**Sequence**
PY17X_1465200 5UTR F	5' - ATAAGCTTGTTTGTATCTTGGATTTTGAAATGGTTG - 3'
PY17X_1465200 5UTR R	5' - AATAGCATTGACCAAGCTAGCTATAGGCGCGCCTTTTGGATCAAACGTGGGGTATATATG - 3'
PY17X_1465200 3UTR F	5' - TCCAAAAGGCGCGCCTATAGCTAGCTTGGTCAATGCTATTATATATTTTTATTACTTCCC - 3'
PY17X_1465200 3UTR R	5' - TAGCGGCCGCTAACCATTACATATTCGATAATGTAAACAG - 3'
PY17X_1465200 guide F	5' - TATTCGATGGAGTTGTCTCTGCTT - 3'
PY17X_1465200 guide R	5' - AAACAAGCAGAGACAACTCCATCG - 3'
P1	5' - AGGCGCGCCTATACCCGGGTTGG - 3'
P2	5' - GCAATAAAATAAGCAGAATGCGTATGC - 3'
P3	5' - ACAATAATACTCCAACCATCTCTTC - 3'
P4	5' - TTTCTCAAAGTTTAAAATAGTATCTCC - 3'
**Primers for the Pf LARC2 generation and genotyping**	
PF3D7_1249700 _Guide 3F	5' - TATTGTTGTTGTTGTTTGCCTTGG - 3'
PF3D7_1249700 _Guide 3R	5' - AAACCCAAGGCAAACAACAACAAC - 3'
PF3D7_1249700 _Guide 5F	5' - TATTATATGTGCTACTCCATATAC - 3'
PF3D7_1249700 _Guide 5R	5' - AAACGTATATGGAGTAGCACATAT - 3'
PF3D7_1249700 _5UTR F	5' - ATACTAGTTTTTATTTTTTATATGAACCATTTAAGAC - 3'
PF3D7_1249700 _5UTR R	5' - TACTTTTCCCCAACCCGGGTATAGGCGCGCCTCTTTCAACAAAAAATAATATATGTGTAC - 3'
PF3D7_1249700 _3UTR F	5' - TTGTTGAAAGAGGCGCGCCTATACCCGGGTTGGGGAAAAGTAAAATTCGCGATAAAAATC - 3'

PF3D7_1249700 _3UTR R	5' - TAGCGGCCGCAATATATATGTGATTTTATTAAGTACGG - 3'
A	5' - TAAAATGATATATGGAATACAATTATG - 3'
B	5' - TTCTTCCTCTTTGATCTTTTTACC - 3'
C	5' - GAAGGCCCATTAAAAAATATAGAC - 3'
D	5' - CCAAGTAACAGAACAGACTTTGTTAC - 3'
E	5'- GGCGCGCCCTTAAGGCCTGCAGG - 3'
F	5' - TAAATGAGGGATAAAACCTCAAAAAG - 3'
G	5' - AATATGTTTCAAACGGTTAAATGTAAG - 3'
H	5' - CTTAACAACCATCTTCTTGACAATAGGG - 3'
I	5' - GTTATGTGATTAACTTTCGCTTGATAC - 3'
J	5' - AGGCGCGCCTATACCCGGGTTGG - 3'
K	5' - CCCAGAAACGCTGGTGAAAG - 3'
L	5' - CGCCTCCATCCAGTCTATTAATTG - 3'
M	5' - CGGAGCCCTGCTGTTCGACAGC - 3'
N	5' - GCCATTCTTCTTCTCGCCGGGCAGCT - 3'
O	5' - CGAATTTAGATATTTCCAGAG - 3'
P	5' - CTTAATGCCTTTCTCCTCCTGG - 3'

Appendix Table S2: PfSPZ-LARC2 liver stages do not transition to productive blood stage infection after sporozoite infection in FRG huHep mice.

Mice	Parasite equivalents/ml (Day 7)	Copies of Pf 18s rRNA/ml (Day 7)	Parasite equivalents/ml (Day 14)	Copies of Pf 18s rRNA/ml (Day 14)
PfSPZ_1	11,518	85,231,282	90372128	668,753,745,913
PfSPZ_2	1,101	8,147,908	159362886	1,179,285,358,246
PfSPZ_3	506,919	3,751,204,288	1045652350	7,737,827,387,488
PfSPZ_4	1,248	9,236,016	734301163	5,433,828,607,469
PfSPZ-LARC2_1	0	1,199	0	0
PfSPZ-LARC2_2	197	1,455,700	10	76,290
PfSPZ-LARC2_3	27	198,041	51	378,030
PfSPZ-LARC2_4	4	32,655	19	141,478
PfSPZ-LARC2_5	11	81,921	11	79,393
PfSPZ-LARC2_6	2,689	19,900,587	58	427,541
