# Supplementary figures and images for: A replication competent Plasmodium falciparum parasite completely attenuated by dual gene deletion
Source: EMBO Mol Med. 2024 Mar 21;16(4):10. doi: 10.1038/s44321-024-00057-7 (PMC11018819; doi:10.1038/s44321-024-00057-7)

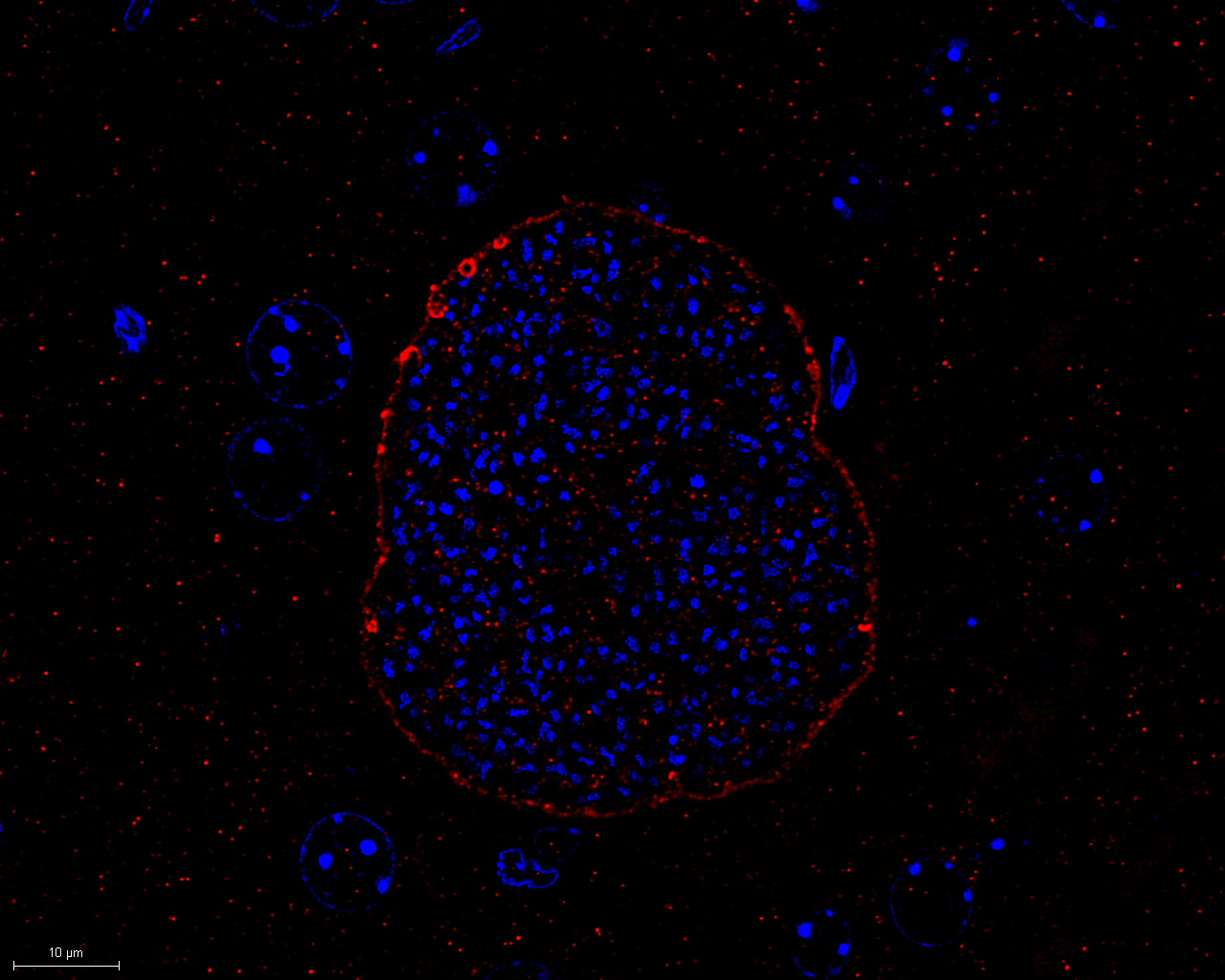

Supplement: Supplementary file 2 — Source Data Fig. 1 [file 44321_2024_57_MOESM2_ESM.zip › Fig 1/1B/PyWT_UIS4DAPI_48h_1.tif]

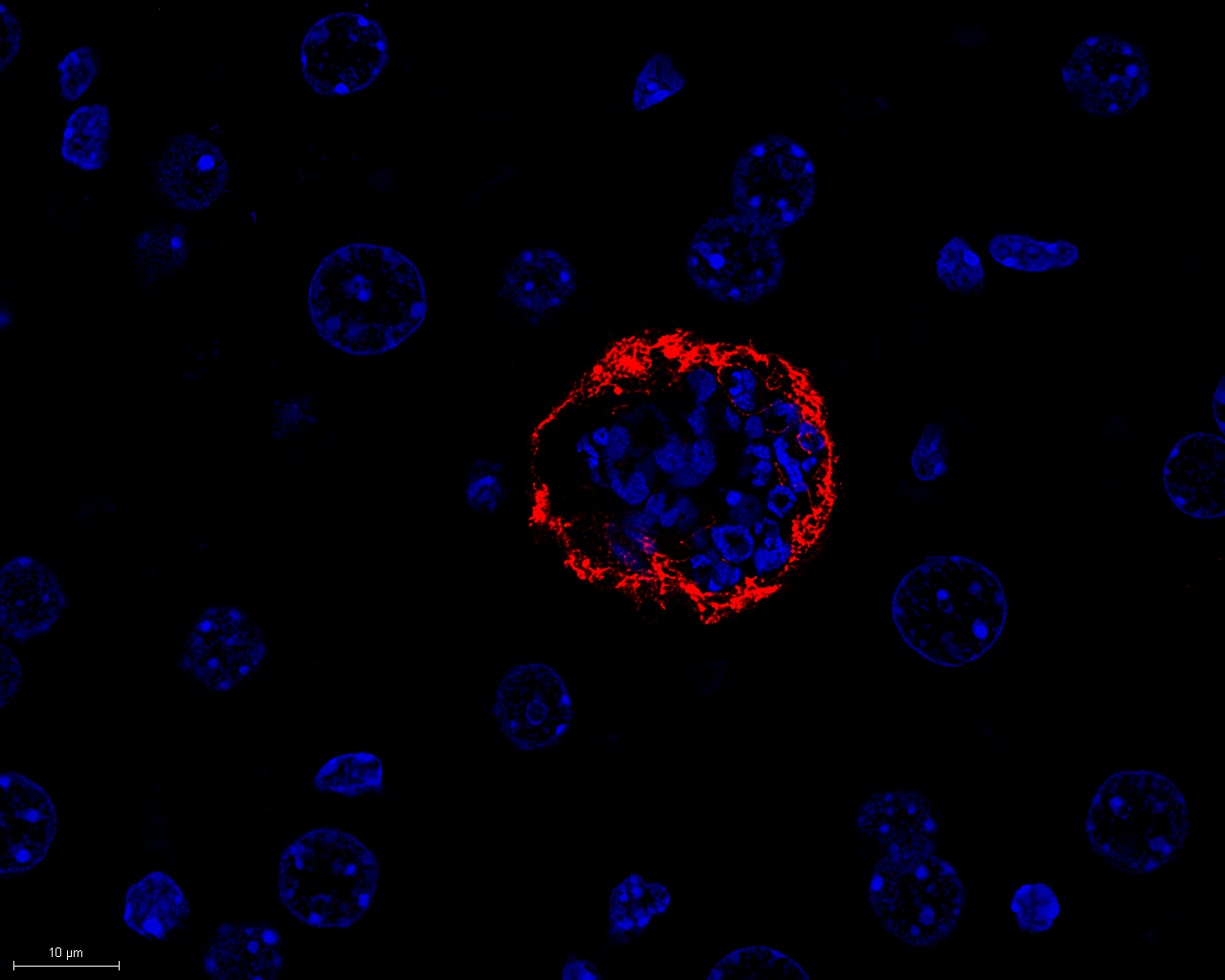

Supplement: Supplementary file 2 — Source Data Fig. 1 [file 44321_2024_57_MOESM2_ESM.zip › Fig 1/1B/PyLARC2_UIS4DAPI_48h_4.tif]

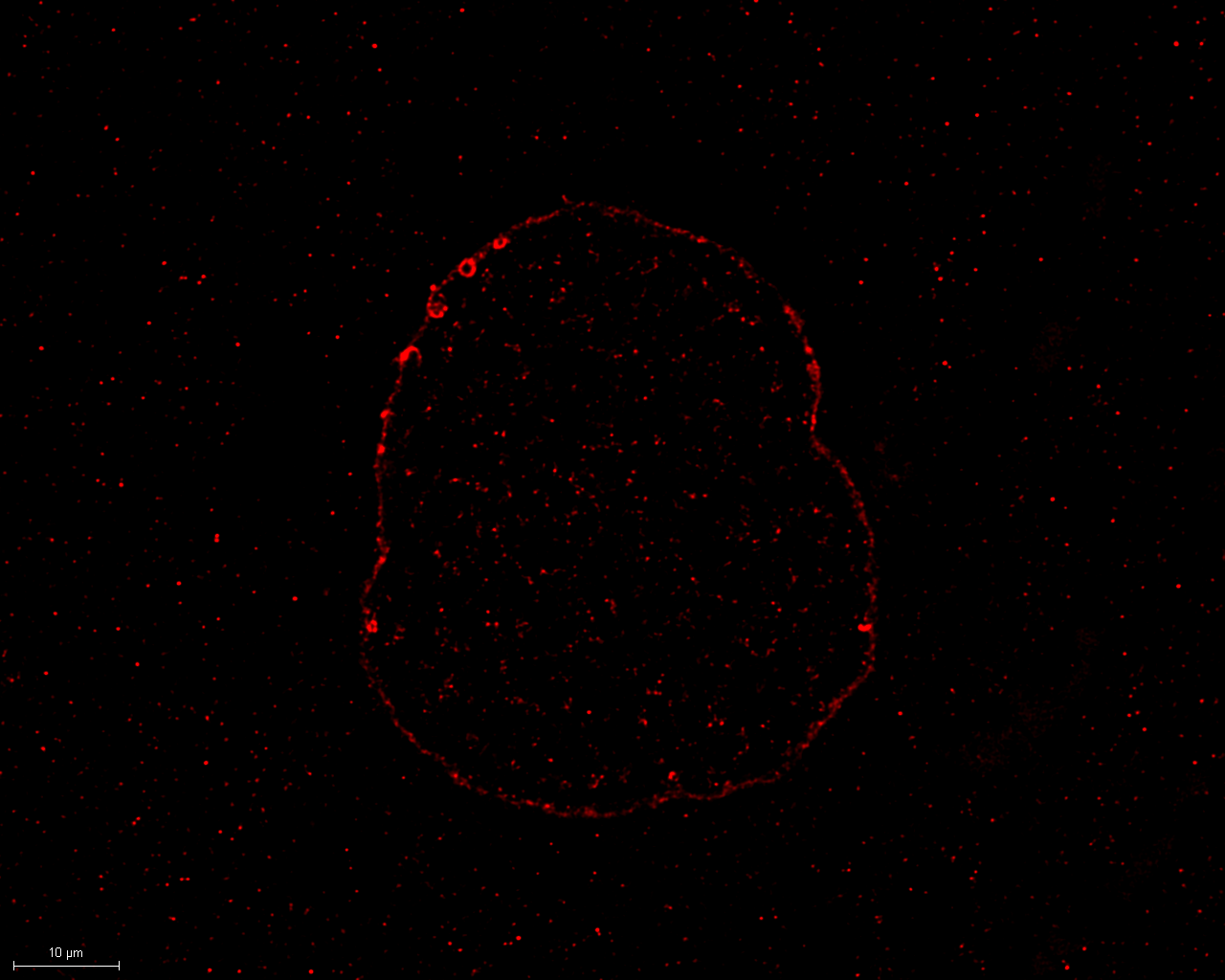

Supplement: Supplementary file 2 — Source Data Fig. 1 [file 44321_2024_57_MOESM2_ESM.zip › Fig 1/1B/PyWT_UIS4DAPI_48h_1_uis4.tif]

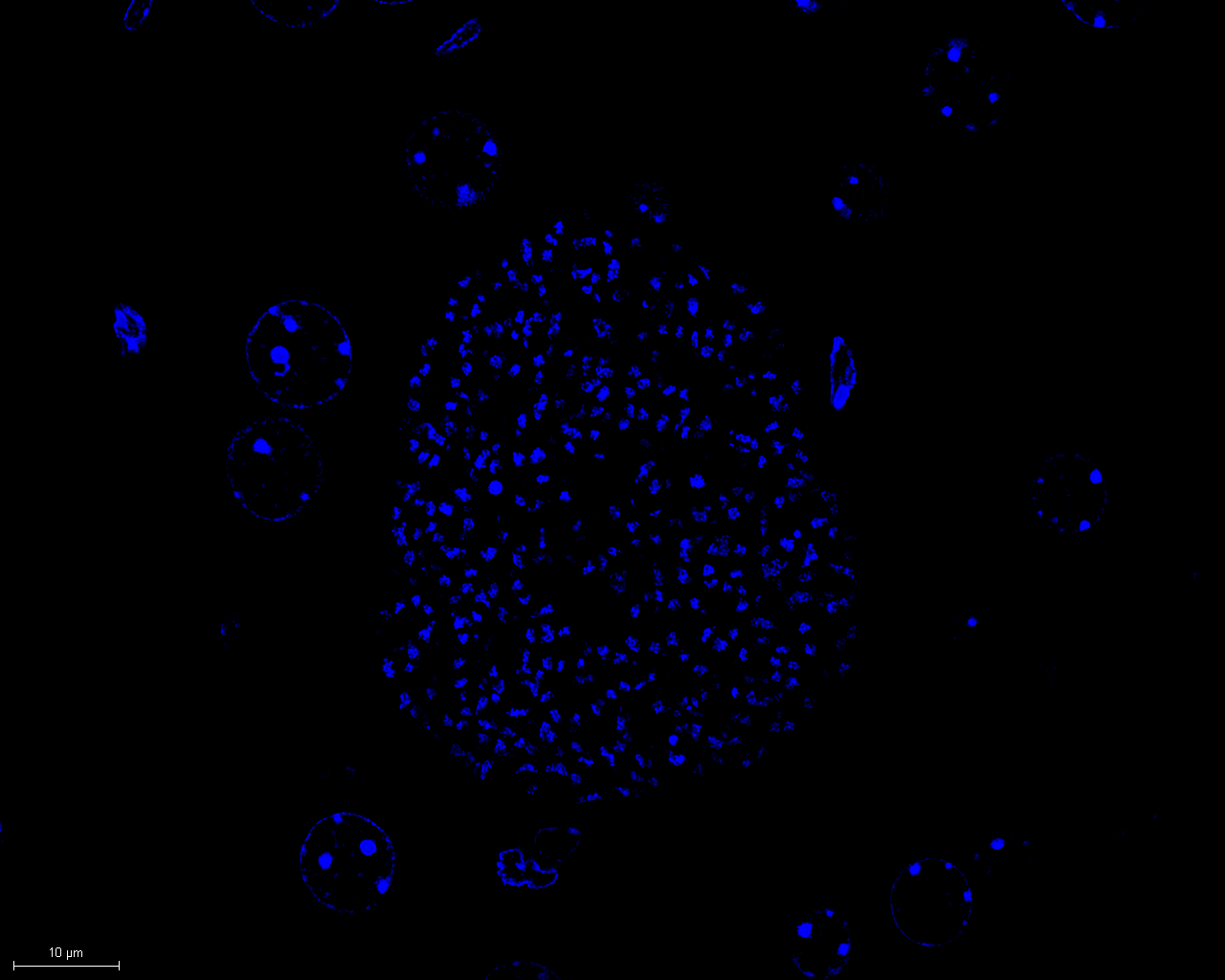

Supplement: Supplementary file 2 — Source Data Fig. 1 [file 44321_2024_57_MOESM2_ESM.zip › Fig 1/1B/PyWT_UIS4DAPI_48h_1_dapi.tif]

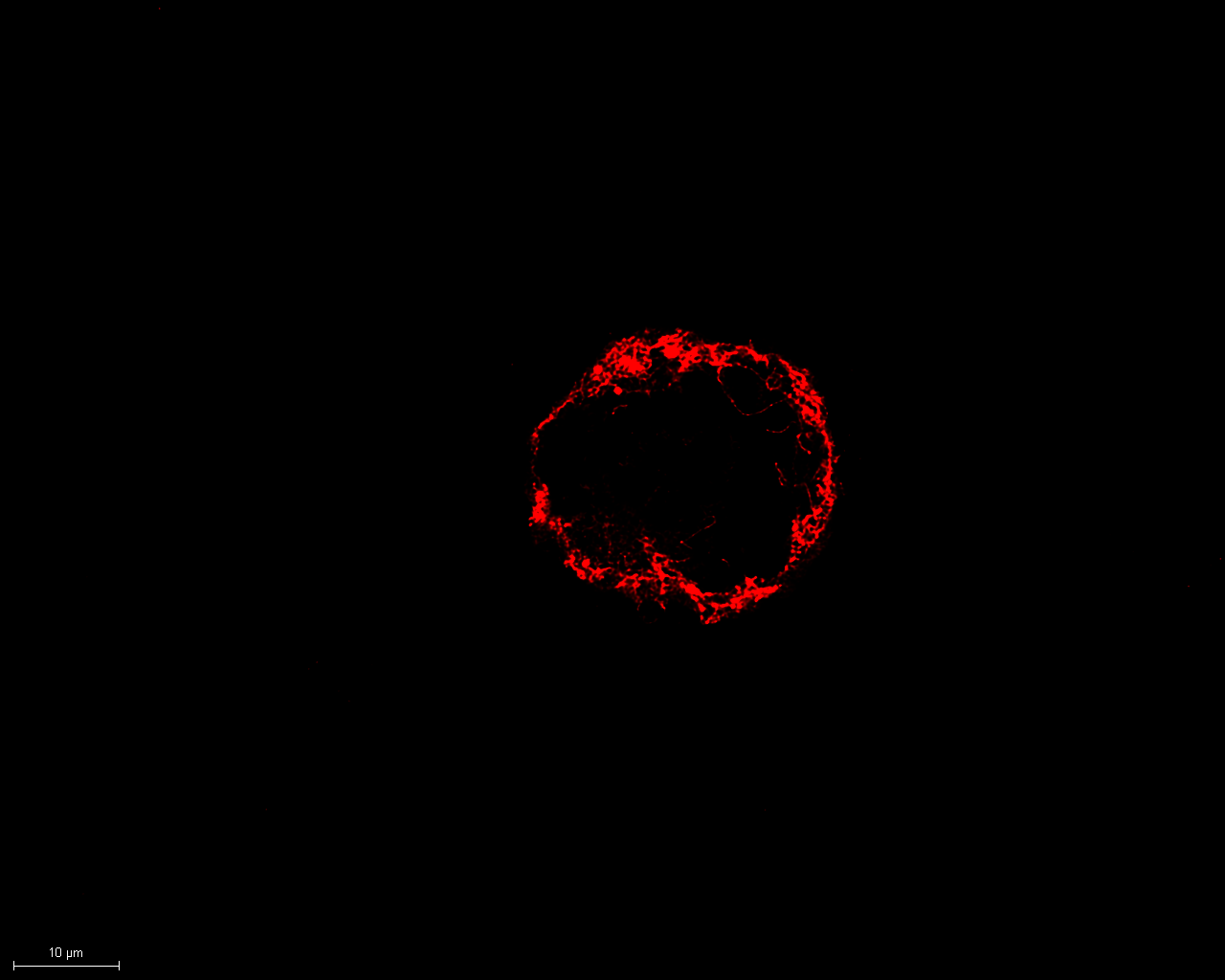

Supplement: Supplementary file 2 — Source Data Fig. 1 [file 44321_2024_57_MOESM2_ESM.zip › Fig 1/1B/PyLARC2_UIS4DAPI_48h_4_UIS4.tif]

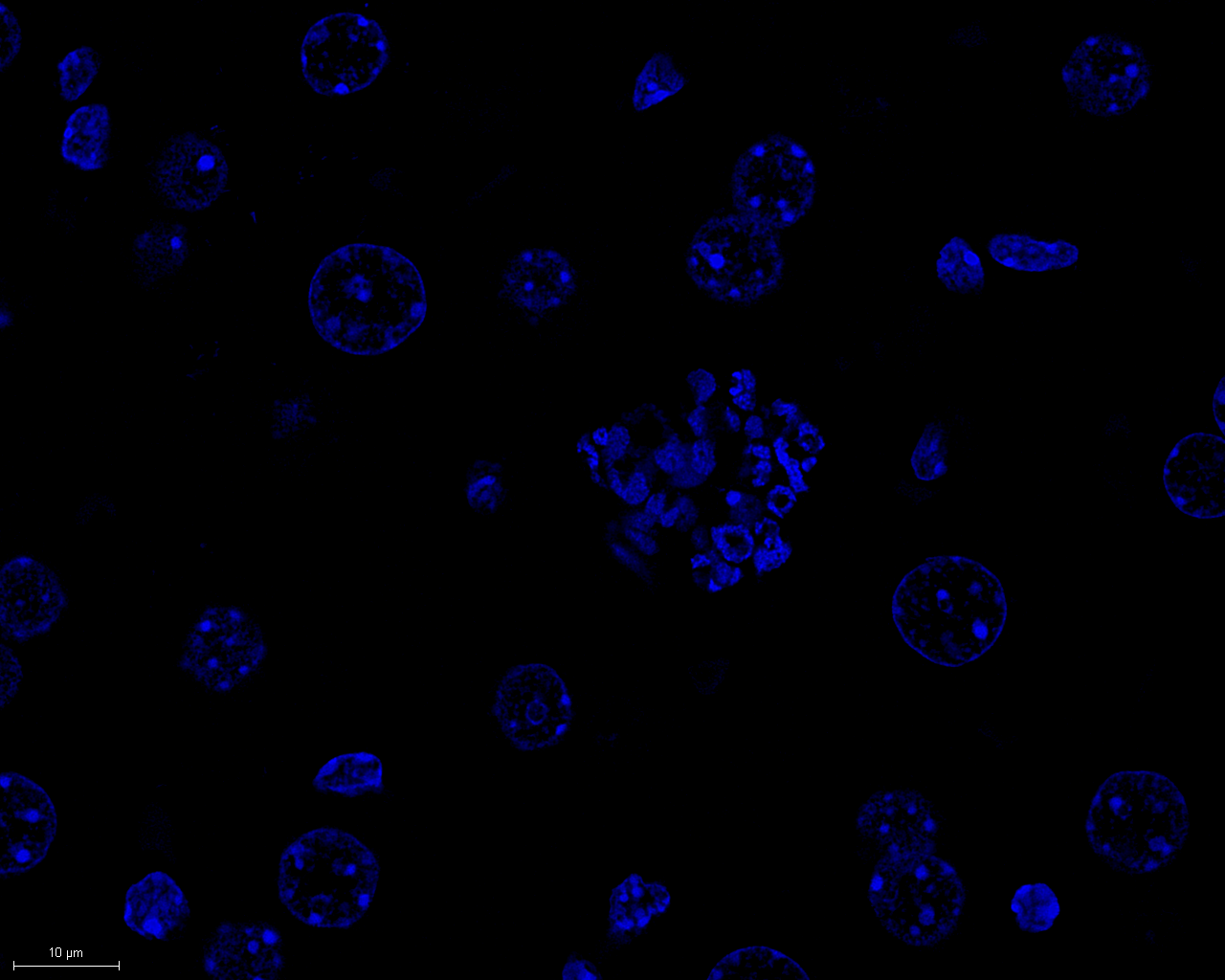

Supplement: Supplementary file 2 — Source Data Fig. 1 [file 44321_2024_57_MOESM2_ESM.zip › Fig 1/1B/PyLARC2_UIS4DAPI_48h_4_dapi.tif]

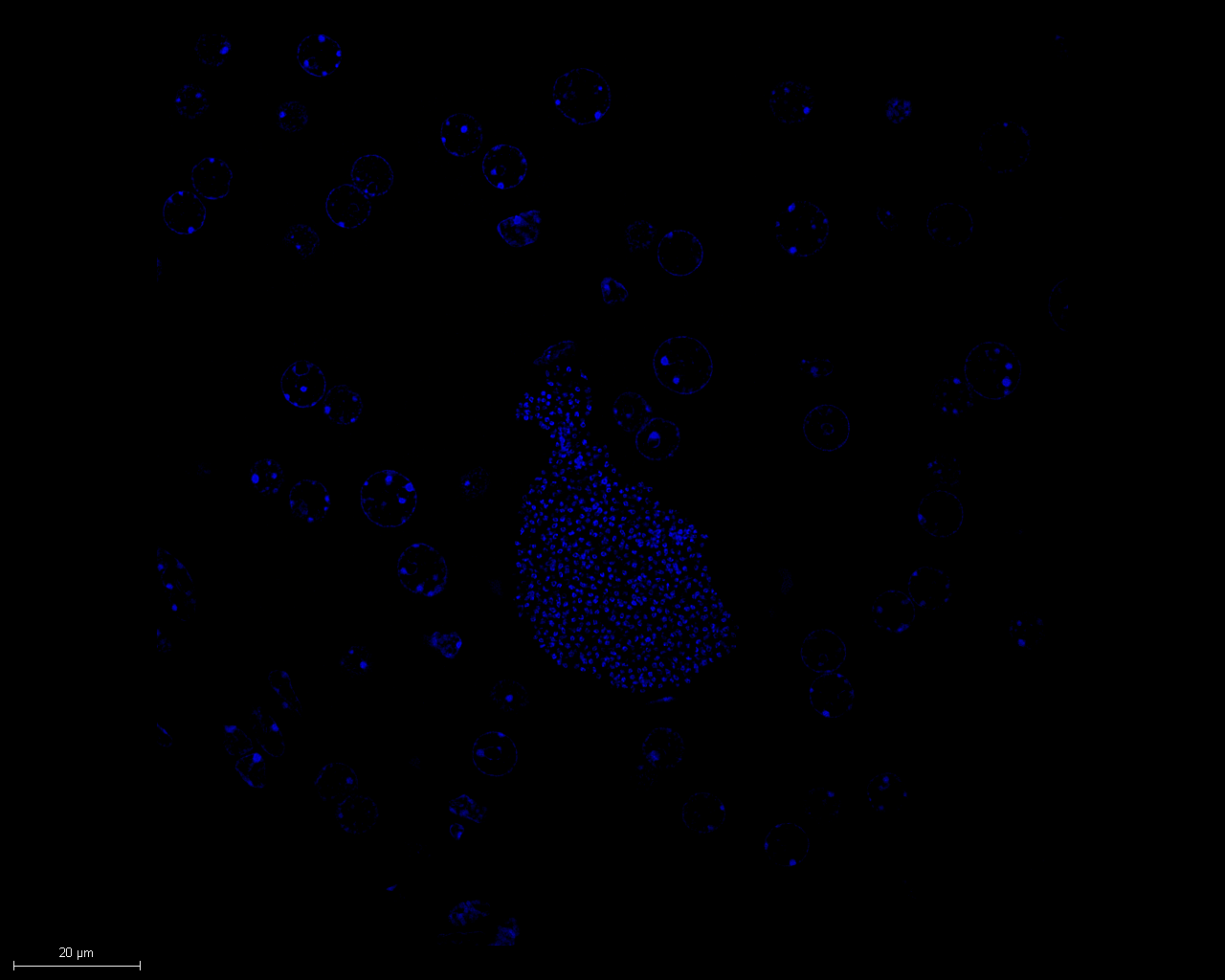

Supplement: Supplementary file 2 — Source Data Fig. 1 [file 44321_2024_57_MOESM2_ESM.zip › Fig 1/1E/PyWT_mTipDAPI_48h_1_dapi.tif]

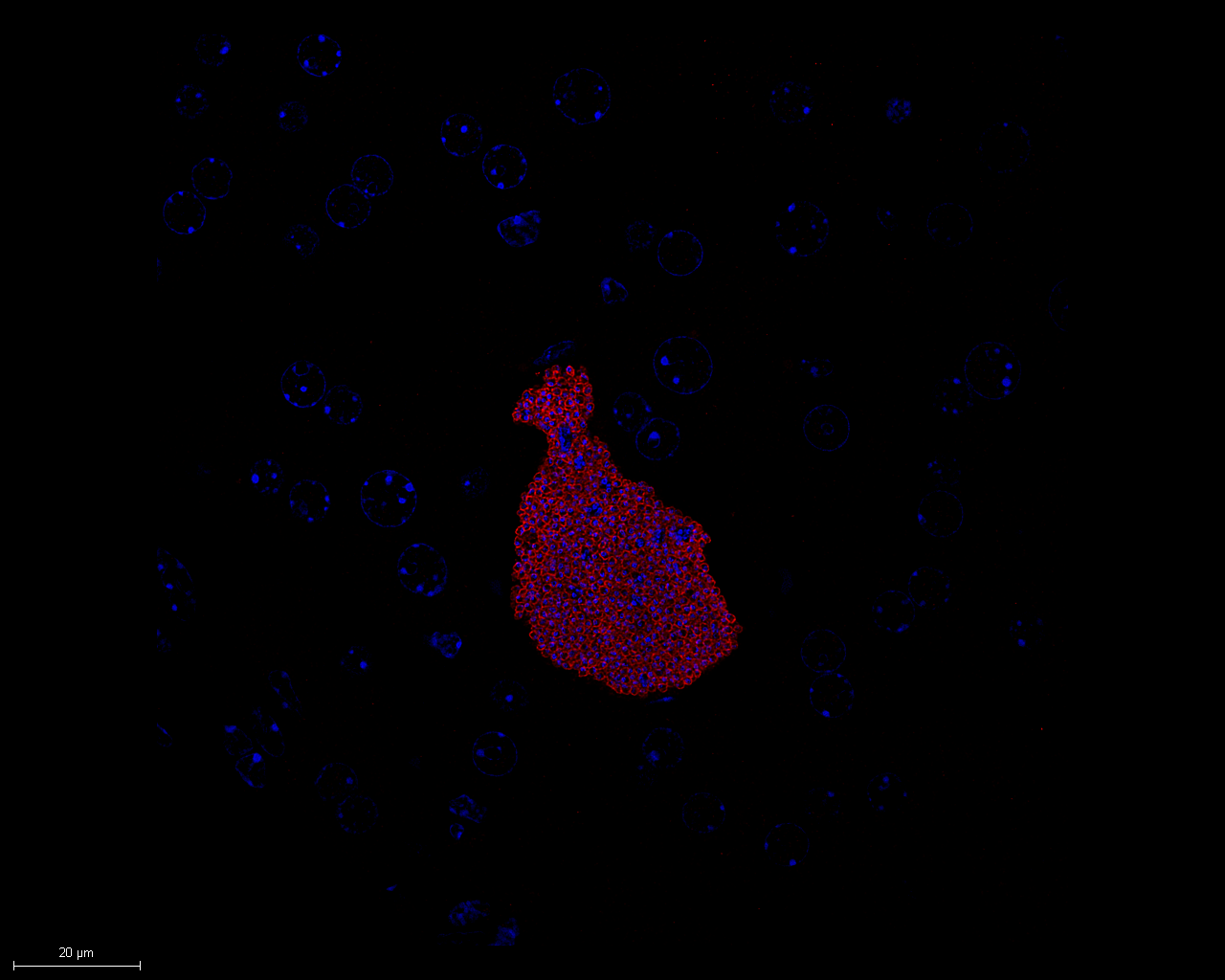

Supplement: Supplementary file 2 — Source Data Fig. 1 [file 44321_2024_57_MOESM2_ESM.zip › Fig 1/1E/PyWT_mTipDAPI_48h_1.tif]

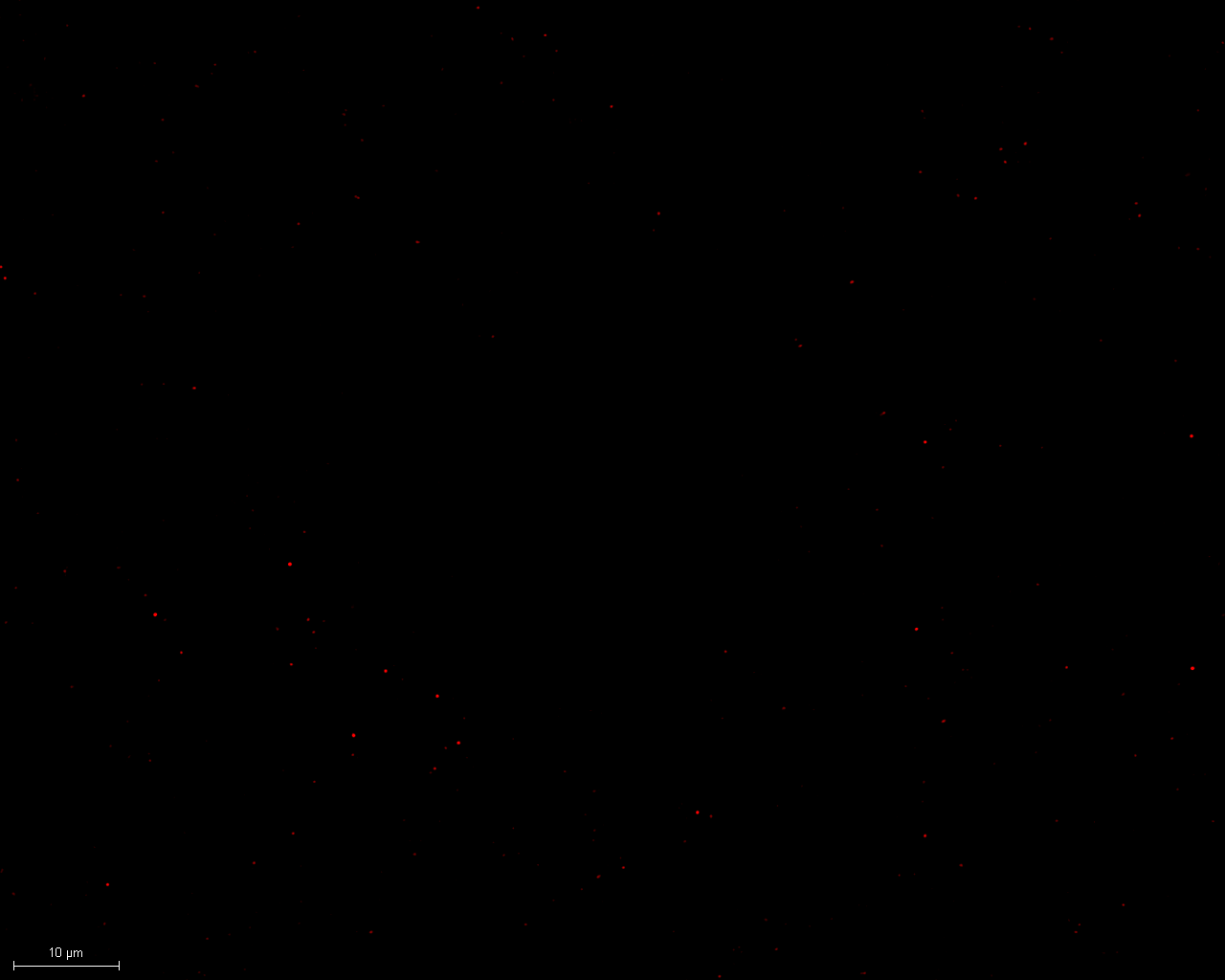

Supplement: Supplementary file 2 — Source Data Fig. 1 [file 44321_2024_57_MOESM2_ESM.zip › Fig 1/1E/PyLACR2_mTipDAPI_48h_1_mTiP.tif]

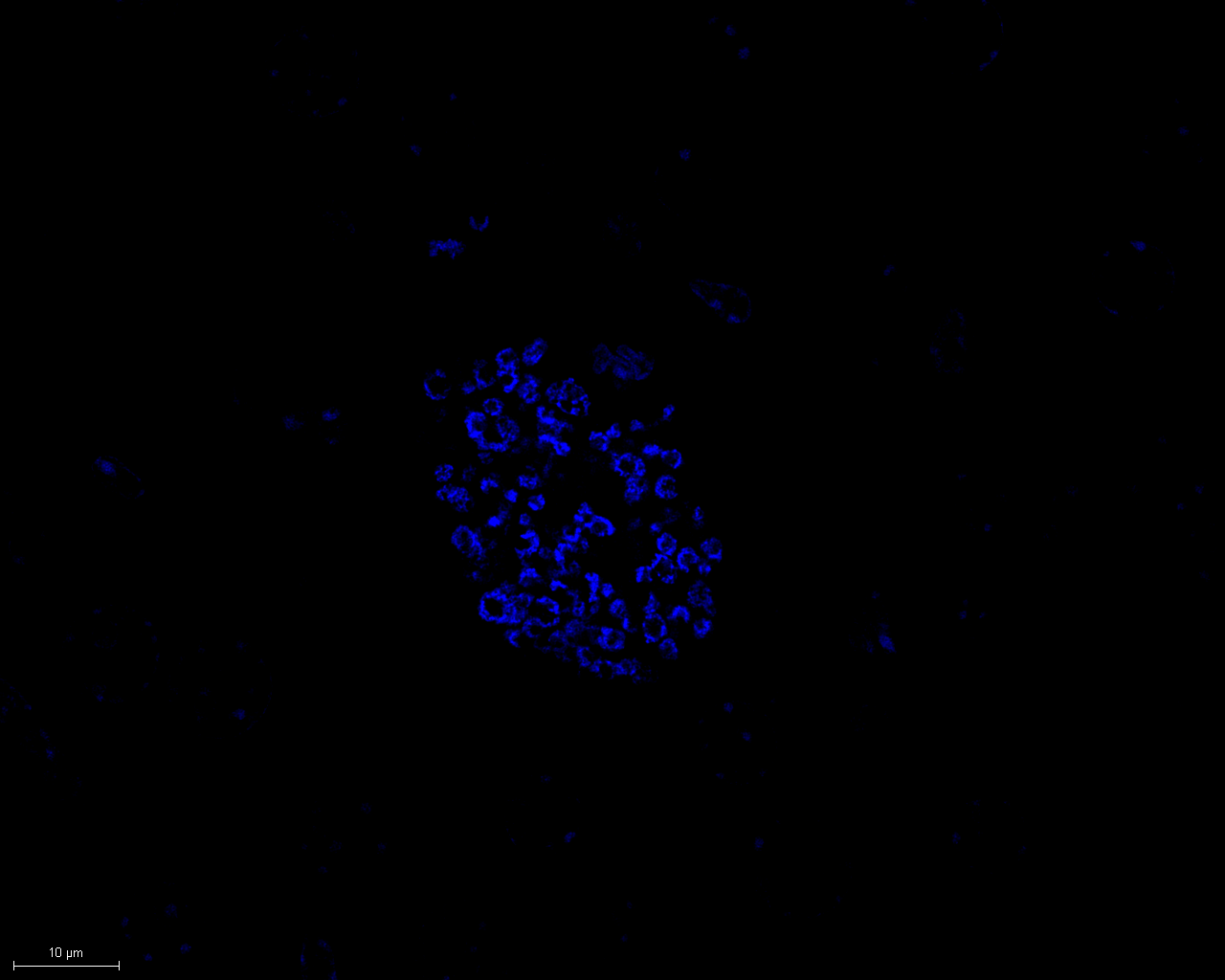

Supplement: Supplementary file 2 — Source Data Fig. 1 [file 44321_2024_57_MOESM2_ESM.zip › Fig 1/1E/PyLACR2_mTipDAPI_48h_1_dapi.tif]

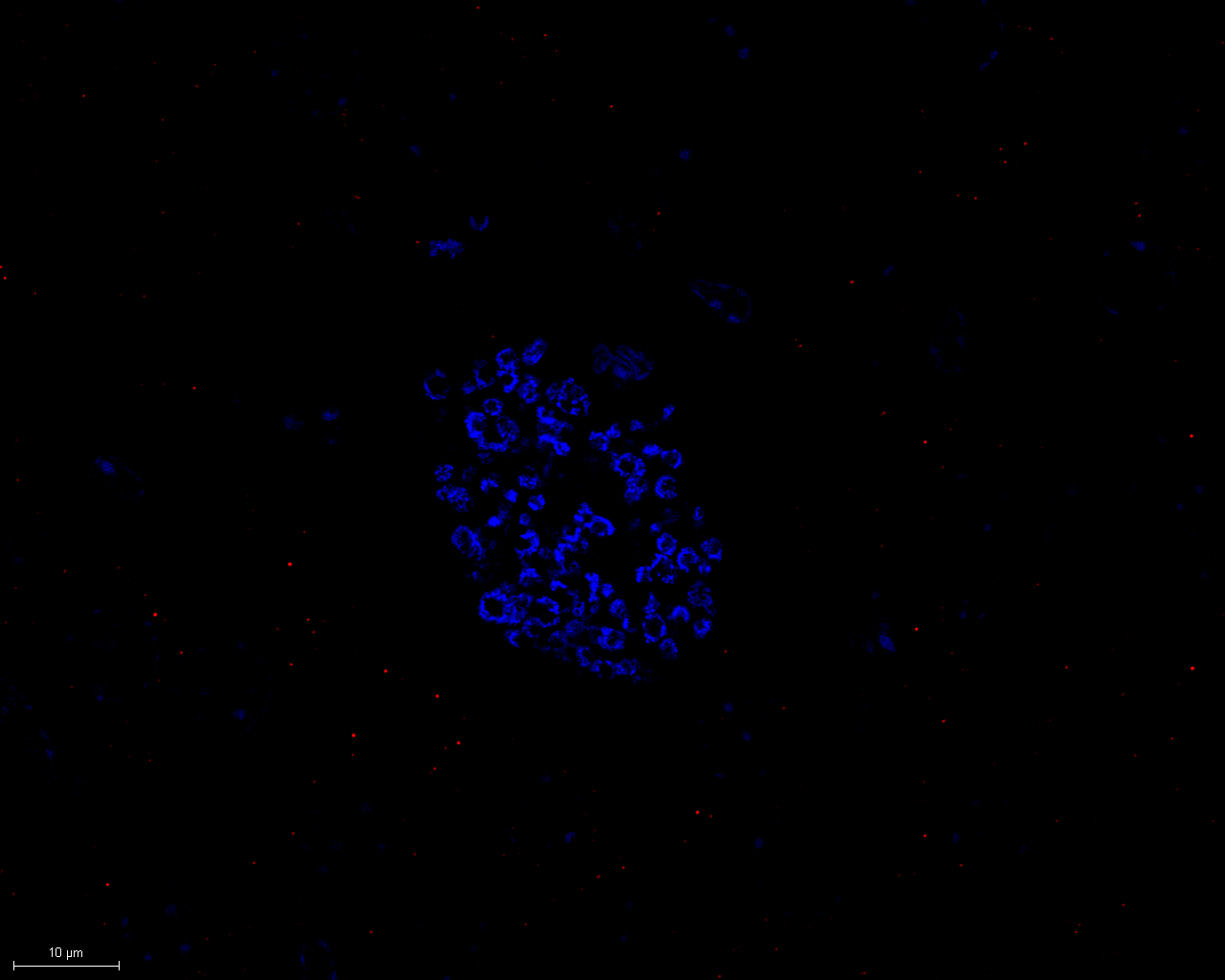

Supplement: Supplementary file 2 — Source Data Fig. 1 [file 44321_2024_57_MOESM2_ESM.zip › Fig 1/1E/PyLACR2_mTipDAPI_48h_1.tif]

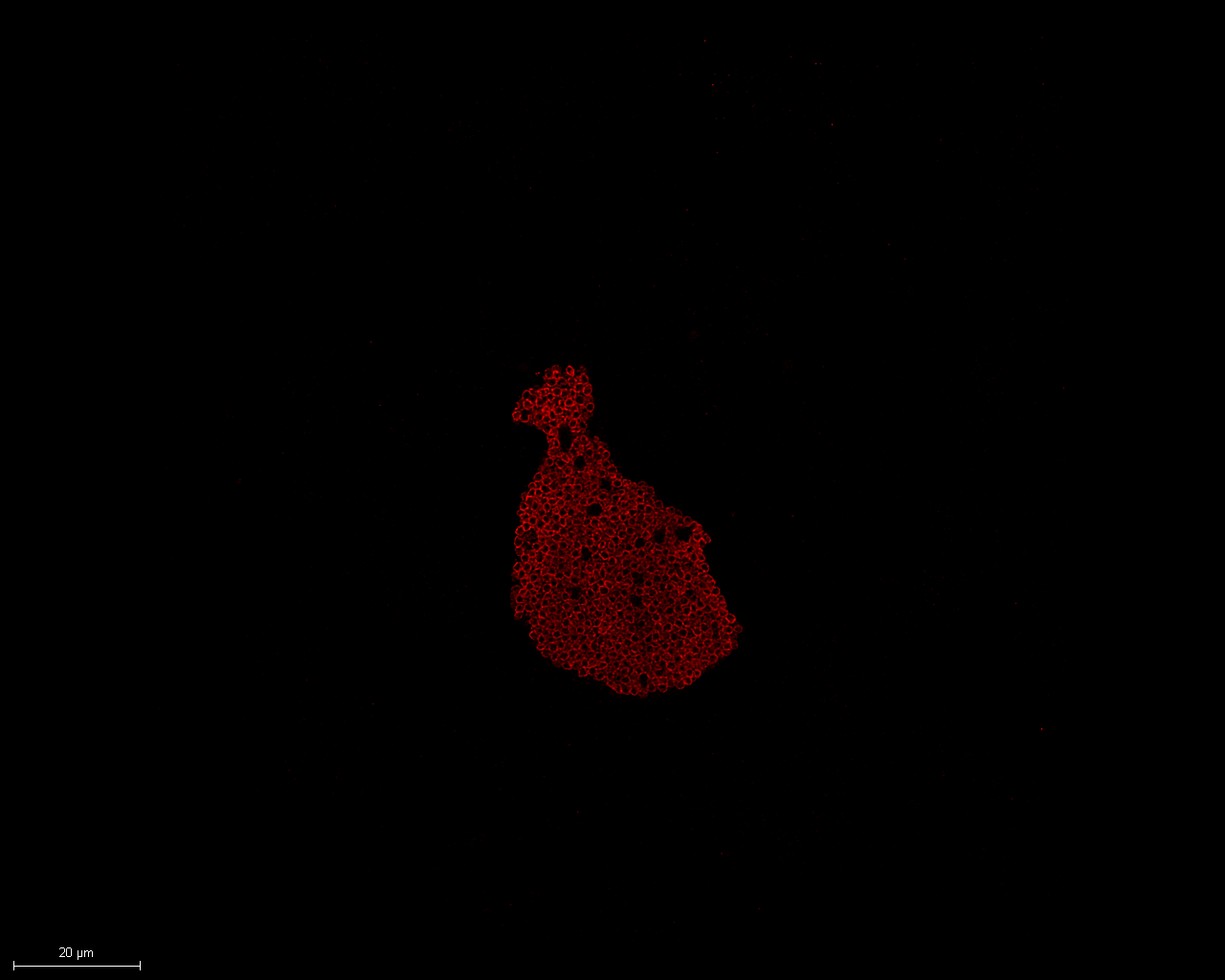

Supplement: Supplementary file 2 — Source Data Fig. 1 [file 44321_2024_57_MOESM2_ESM.zip › Fig 1/1E/PyWT_mTipDAPI_48h_1_mtip.tif]

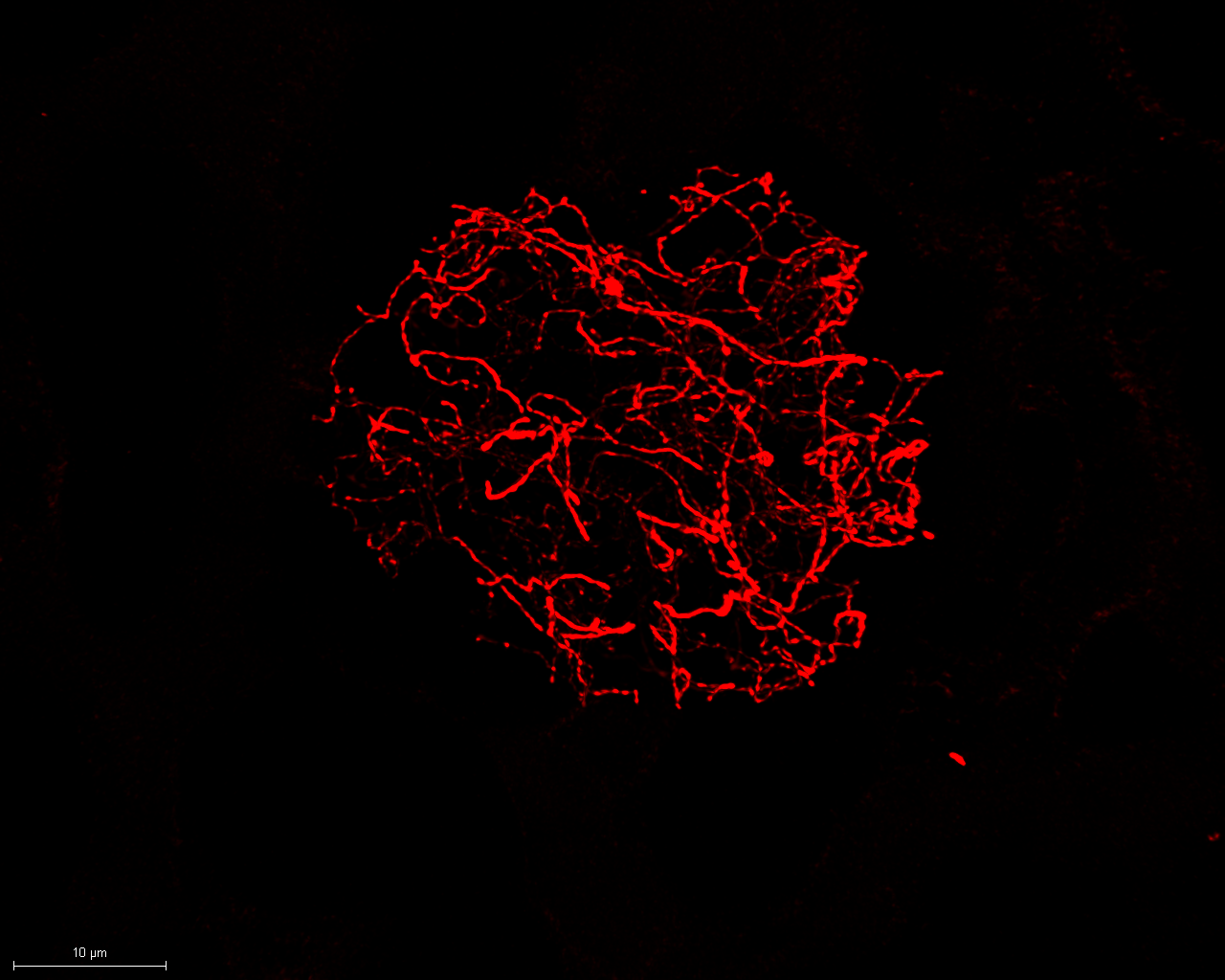

Supplement: Supplementary file 2 — Source Data Fig. 1 [file 44321_2024_57_MOESM2_ESM.zip › Fig 1/1D/larc2_acp-projection.tif]

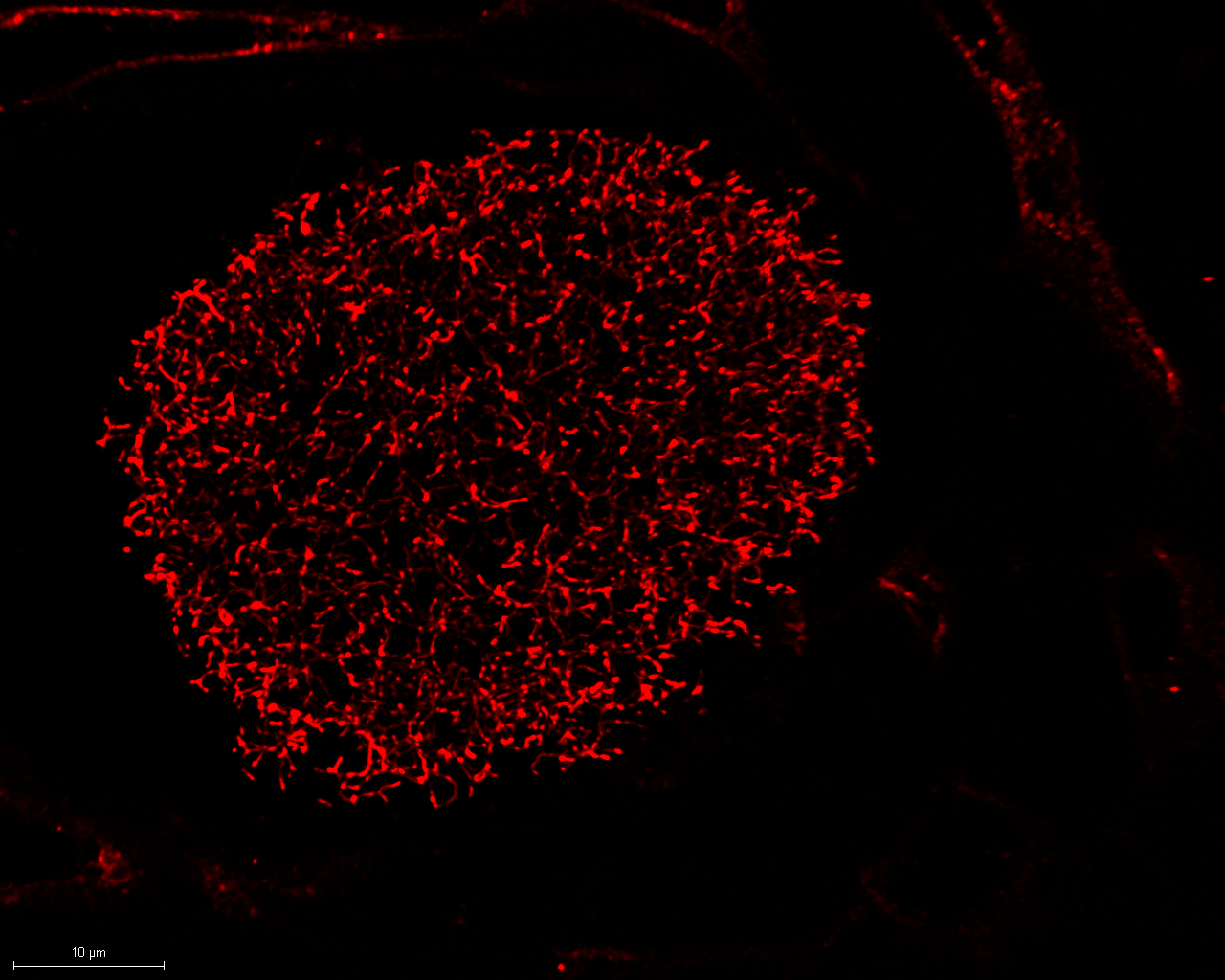

Supplement: Supplementary file 2 — Source Data Fig. 1 [file 44321_2024_57_MOESM2_ESM.zip › Fig 1/1D/wt_acp-projection.tif]

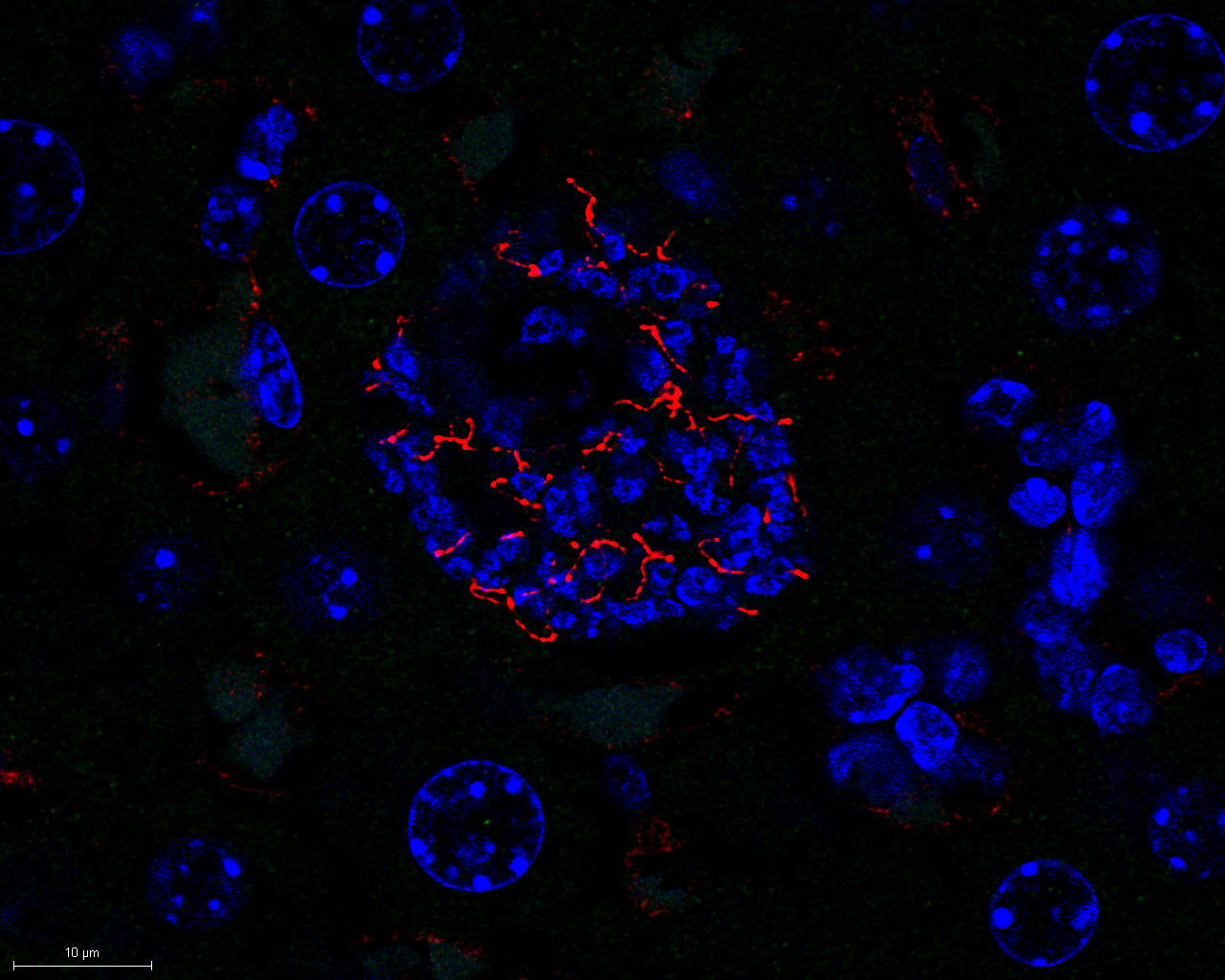

Supplement: Supplementary file 2 — Source Data Fig. 1 [file 44321_2024_57_MOESM2_ESM.zip › Fig 1/1C/larc2_48h_MSP1_Acp_Merge_1.tif]

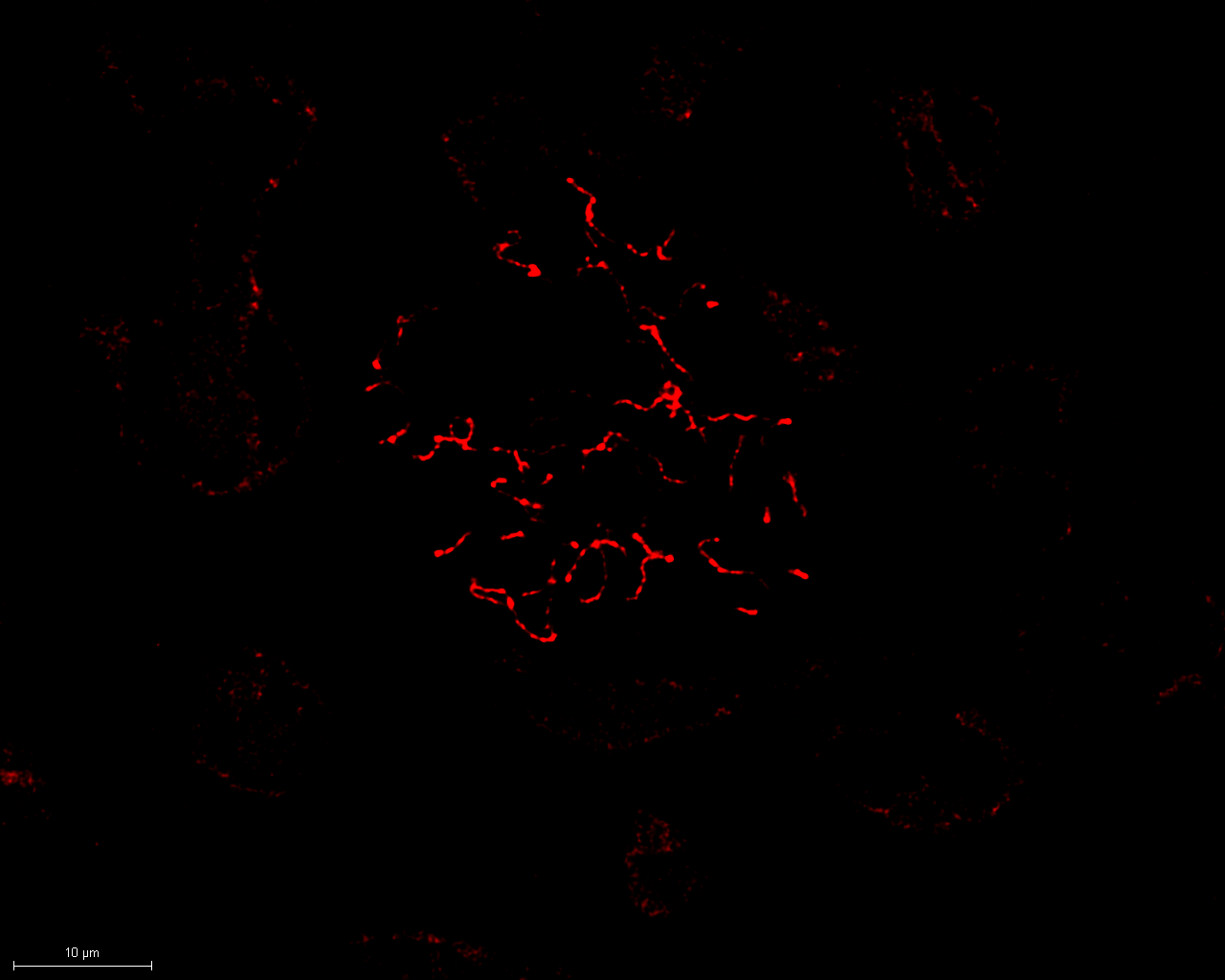

Supplement: Supplementary file 2 — Source Data Fig. 1 [file 44321_2024_57_MOESM2_ESM.zip › Fig 1/1C/larc2_48h_MSP1_Acp_acp_1.tif]

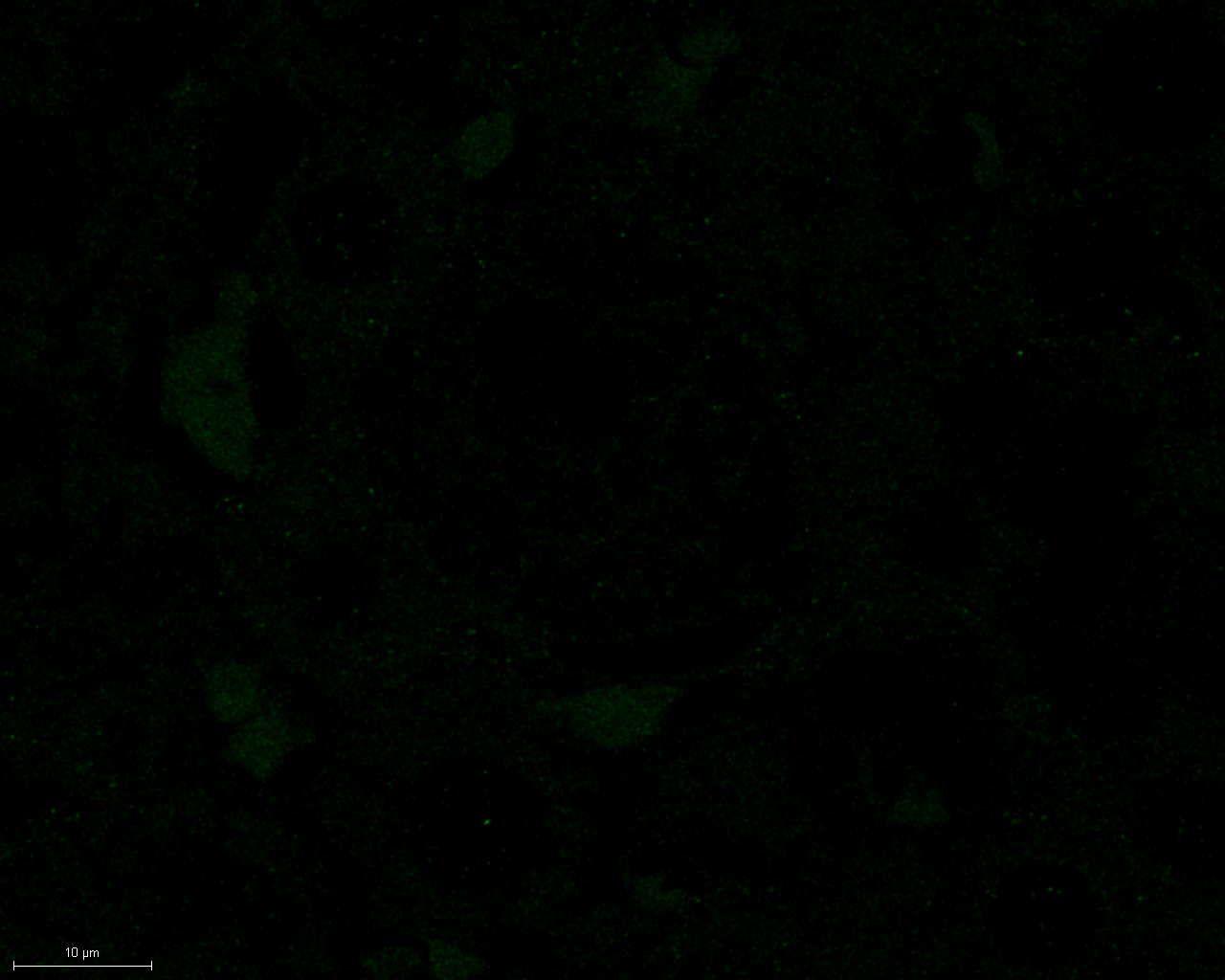

Supplement: Supplementary file 2 — Source Data Fig. 1 [file 44321_2024_57_MOESM2_ESM.zip › Fig 1/1C/larc2_48h_MSP1_Acp_msp1_1.tif]

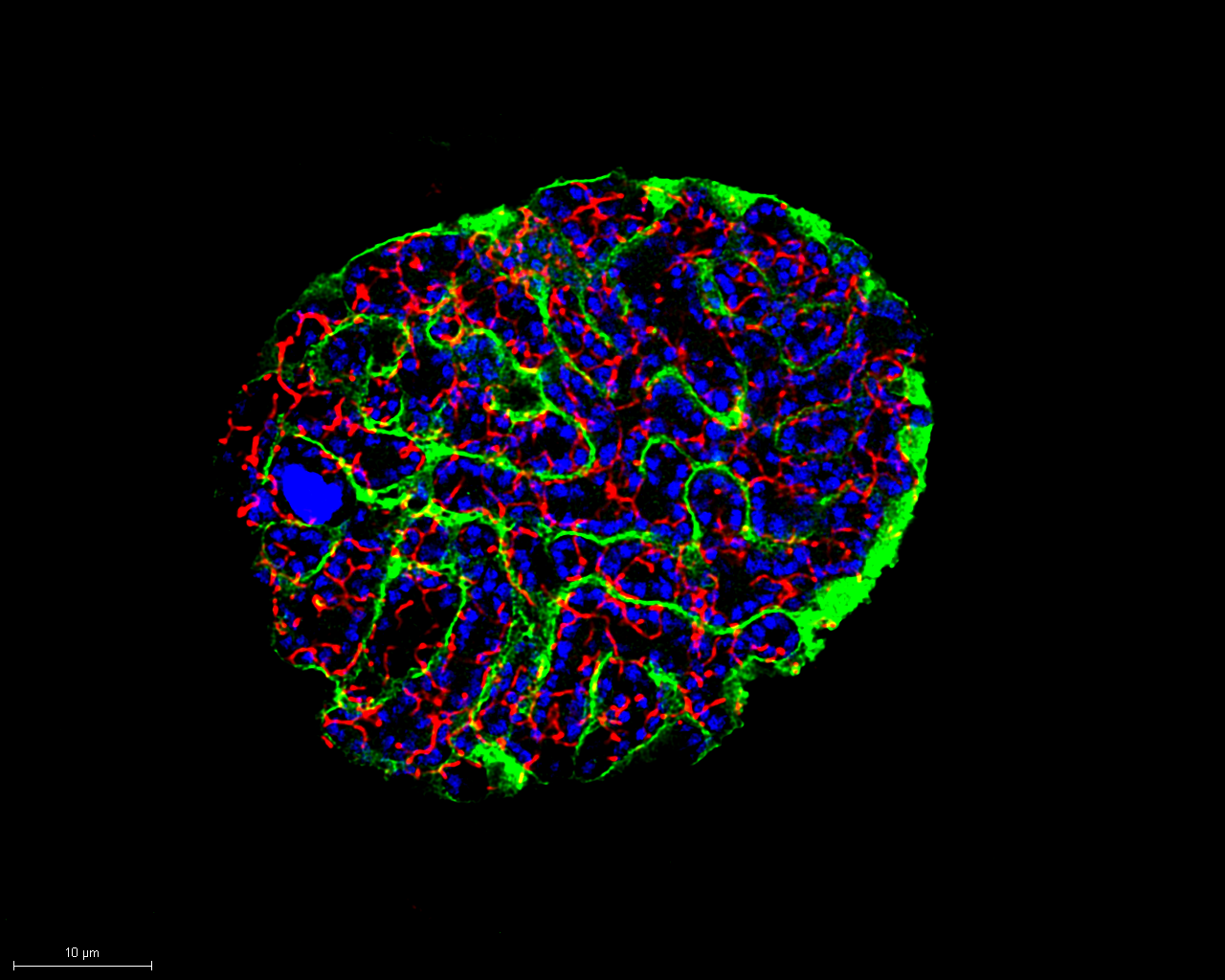

Supplement: Supplementary file 2 — Source Data Fig. 1 [file 44321_2024_57_MOESM2_ESM.zip › Fig 1/1C/wt48h_MSP1_Acp_Merge_2.tif]

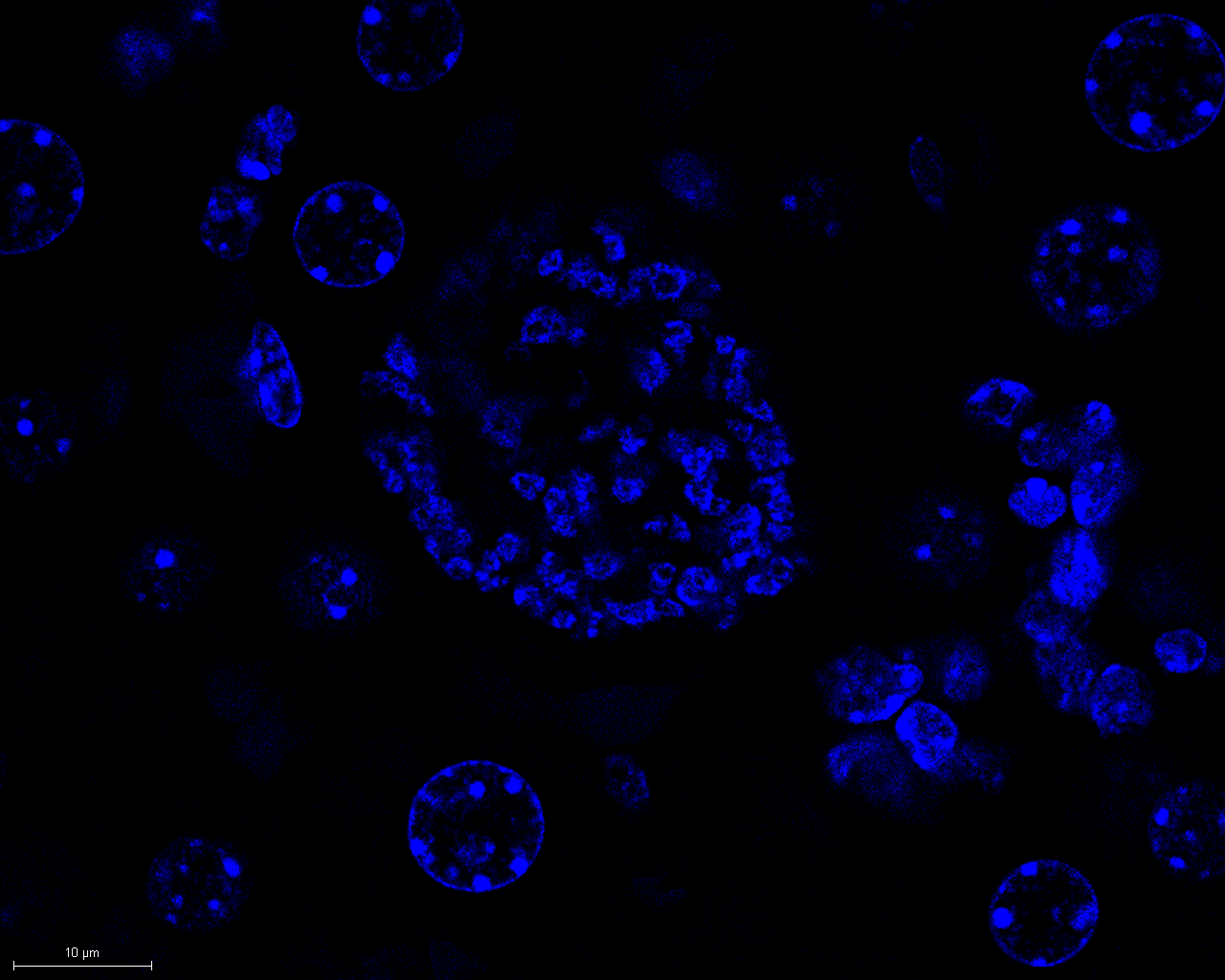

Supplement: Supplementary file 2 — Source Data Fig. 1 [file 44321_2024_57_MOESM2_ESM.zip › Fig 1/1C/larc2_48h_MSP1_Acp_dapi_1.tif]

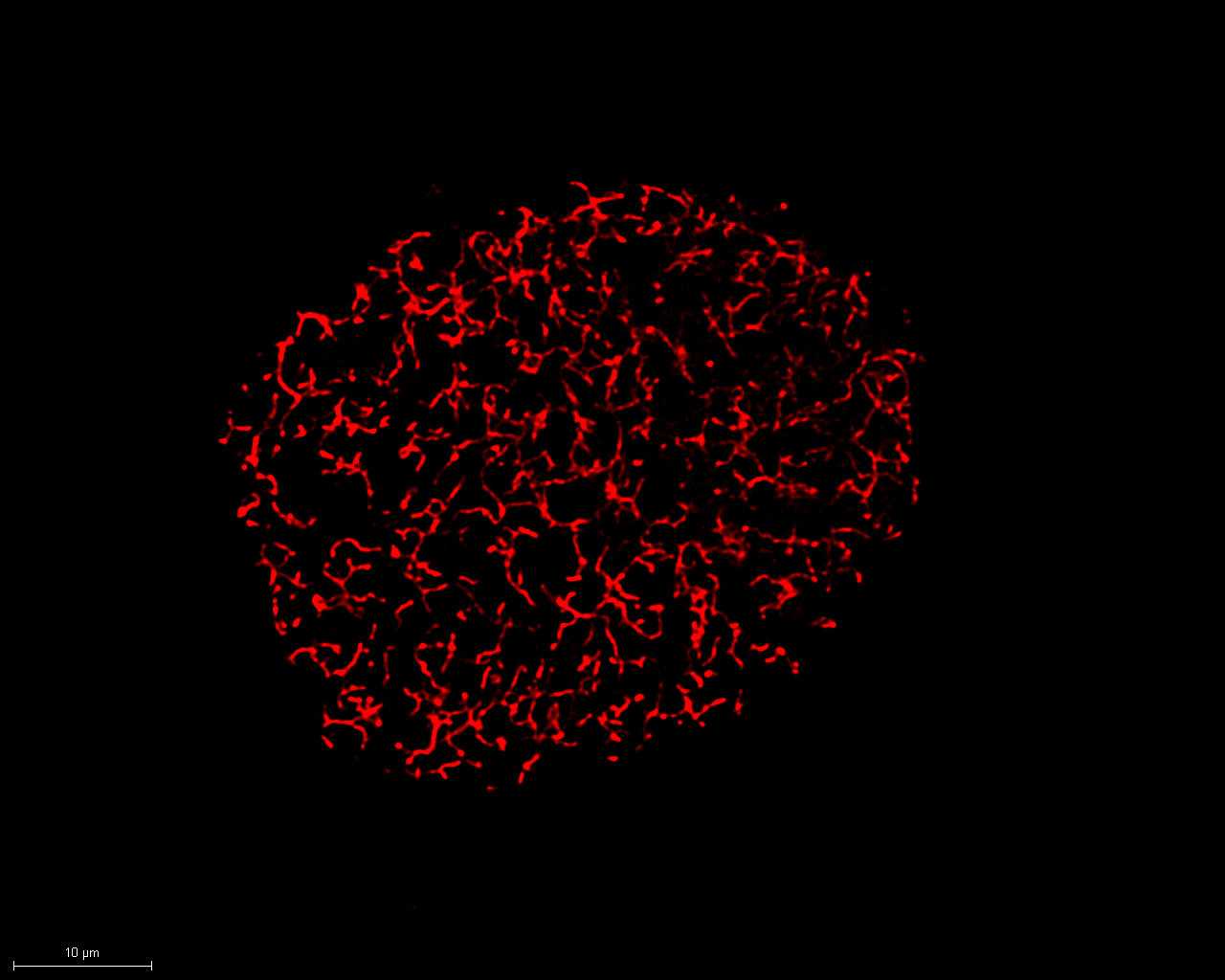

Supplement: Supplementary file 2 — Source Data Fig. 1 [file 44321_2024_57_MOESM2_ESM.zip › Fig 1/1C/wt48h_MSP1_Acp_ACP_2.tif]

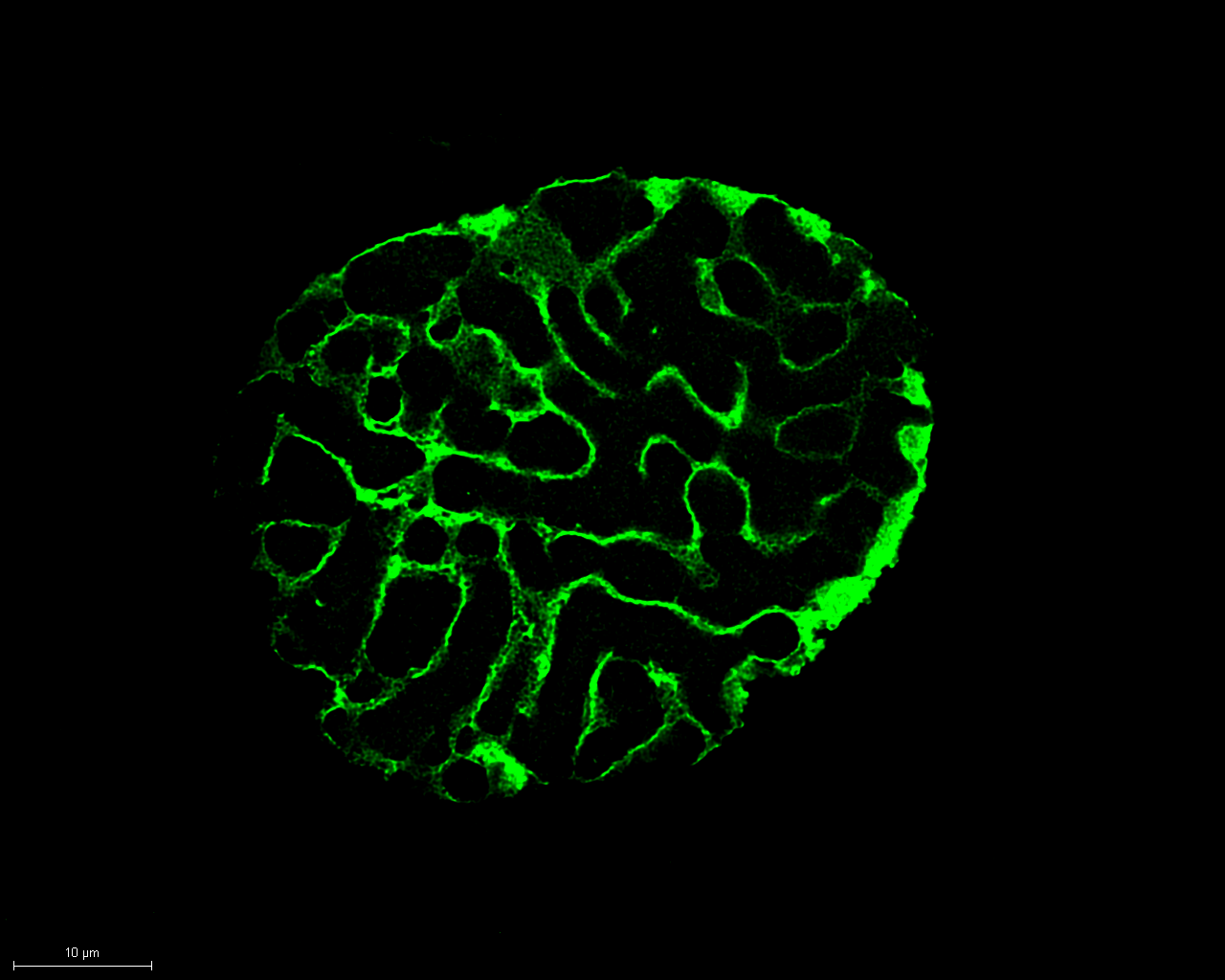

Supplement: Supplementary file 2 — Source Data Fig. 1 [file 44321_2024_57_MOESM2_ESM.zip › Fig 1/1C/wt48h_MSP1_Acp_MSP1_2.tif]

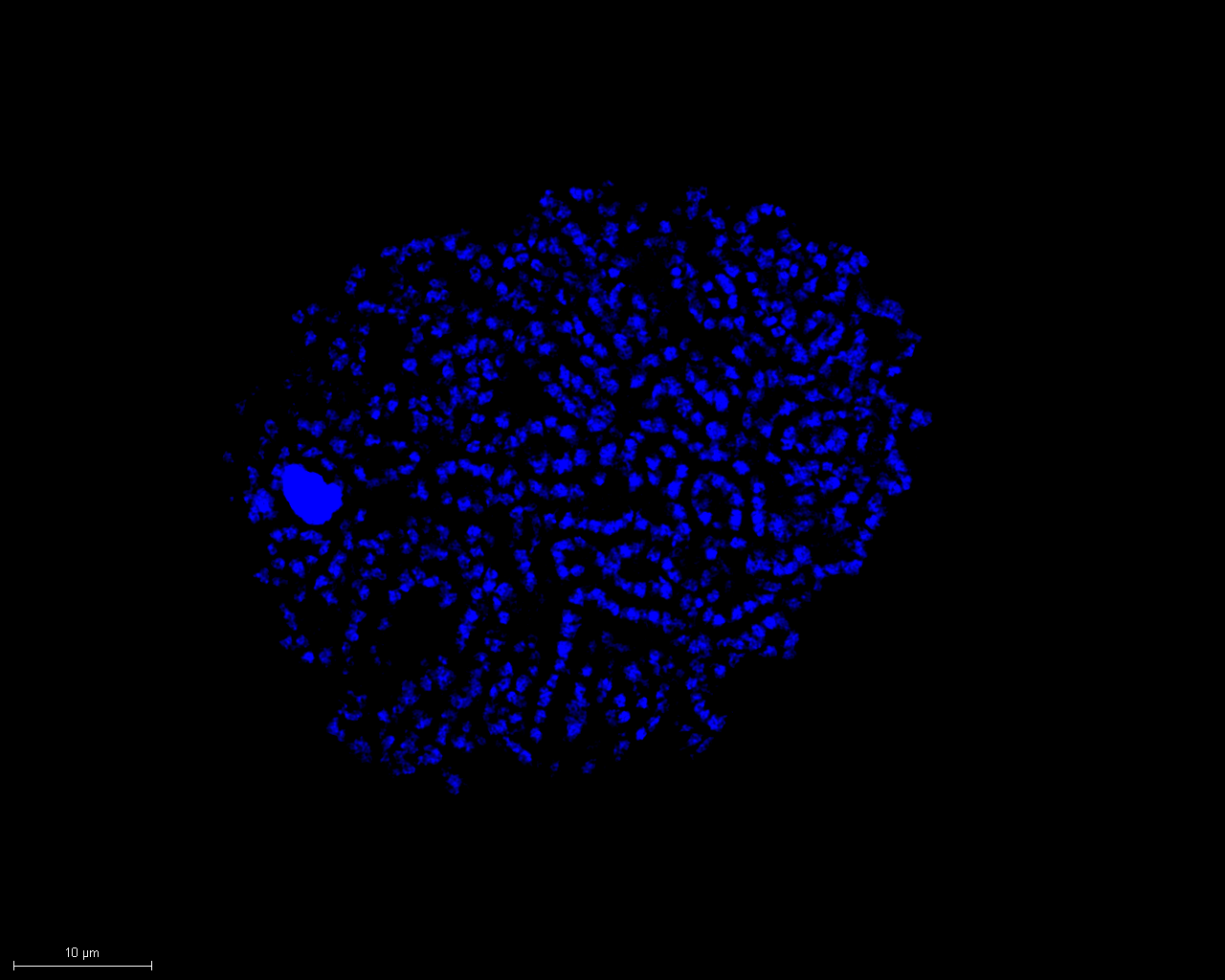

Supplement: Supplementary file 2 — Source Data Fig. 1 [file 44321_2024_57_MOESM2_ESM.zip › Fig 1/1C/wt48h_MSP1_Acp_dapi_2.tif]

## Slide 1
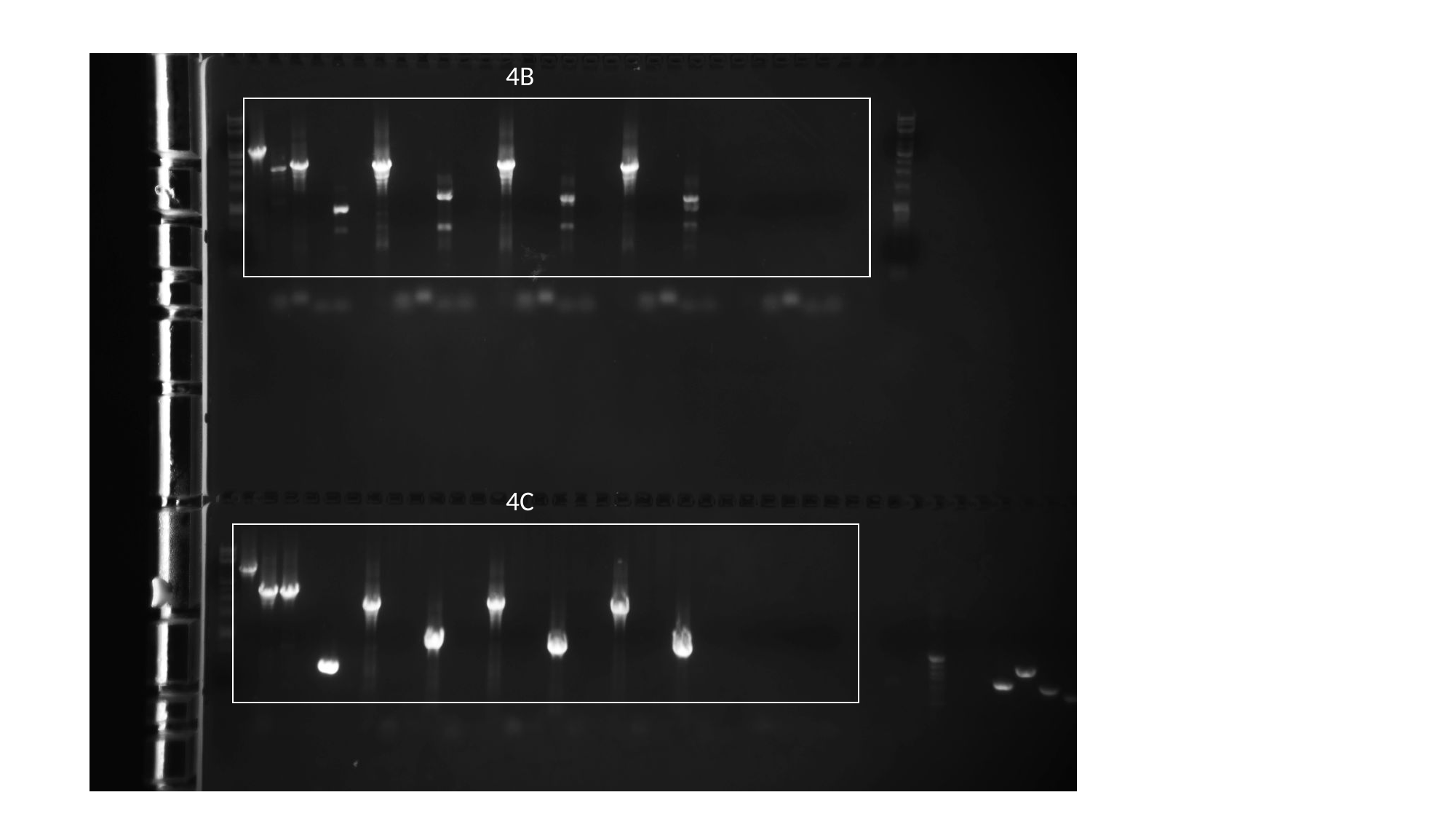

4B
4C

## Slide 2
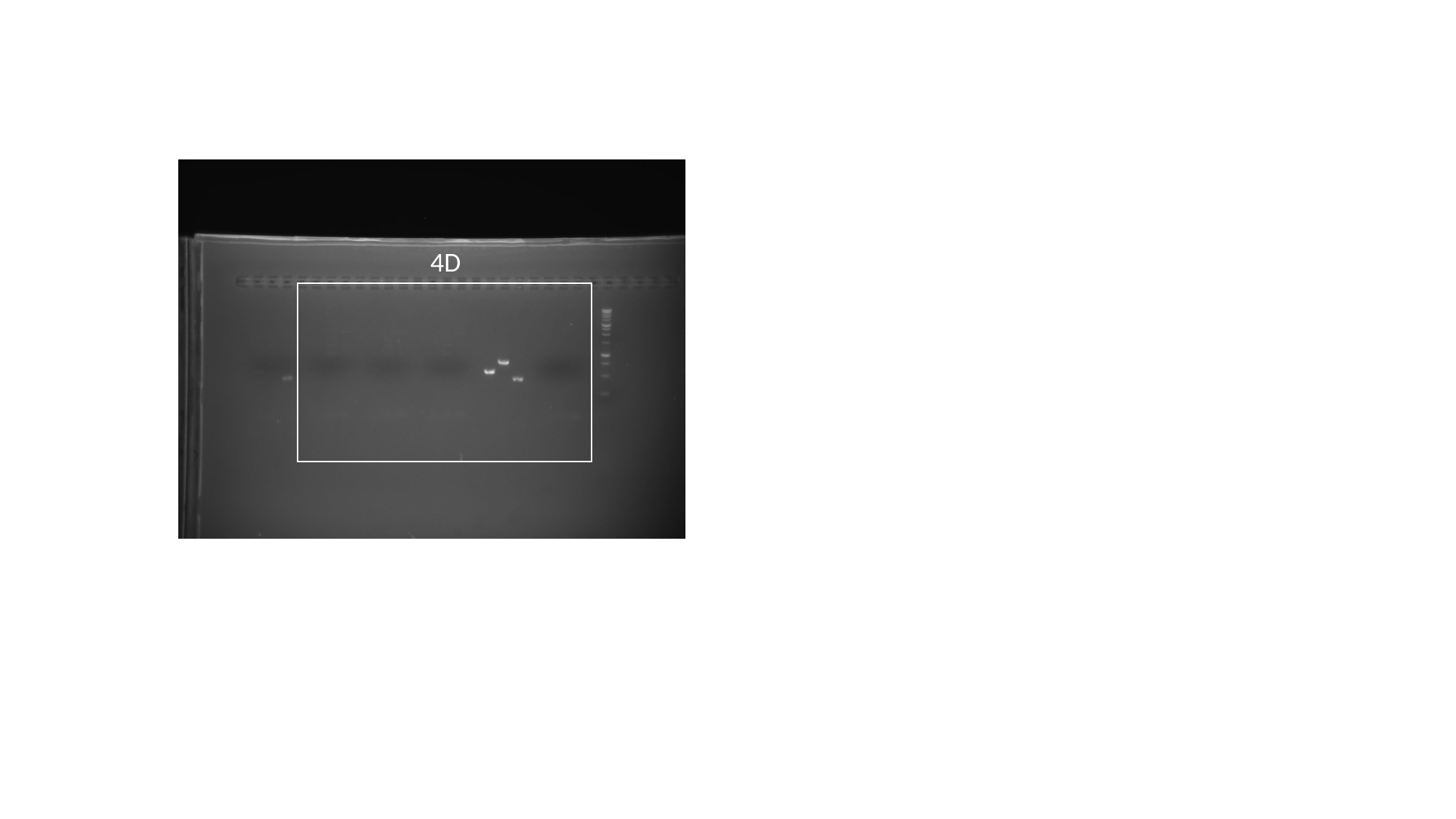

4D

Supplement: Supplementary file 5 — Source Data Fig. 4 [file 44321_2024_57_MOESM5_ESM.zip › Fig 4/Figure 4 gel images with cropping borders.pptx]

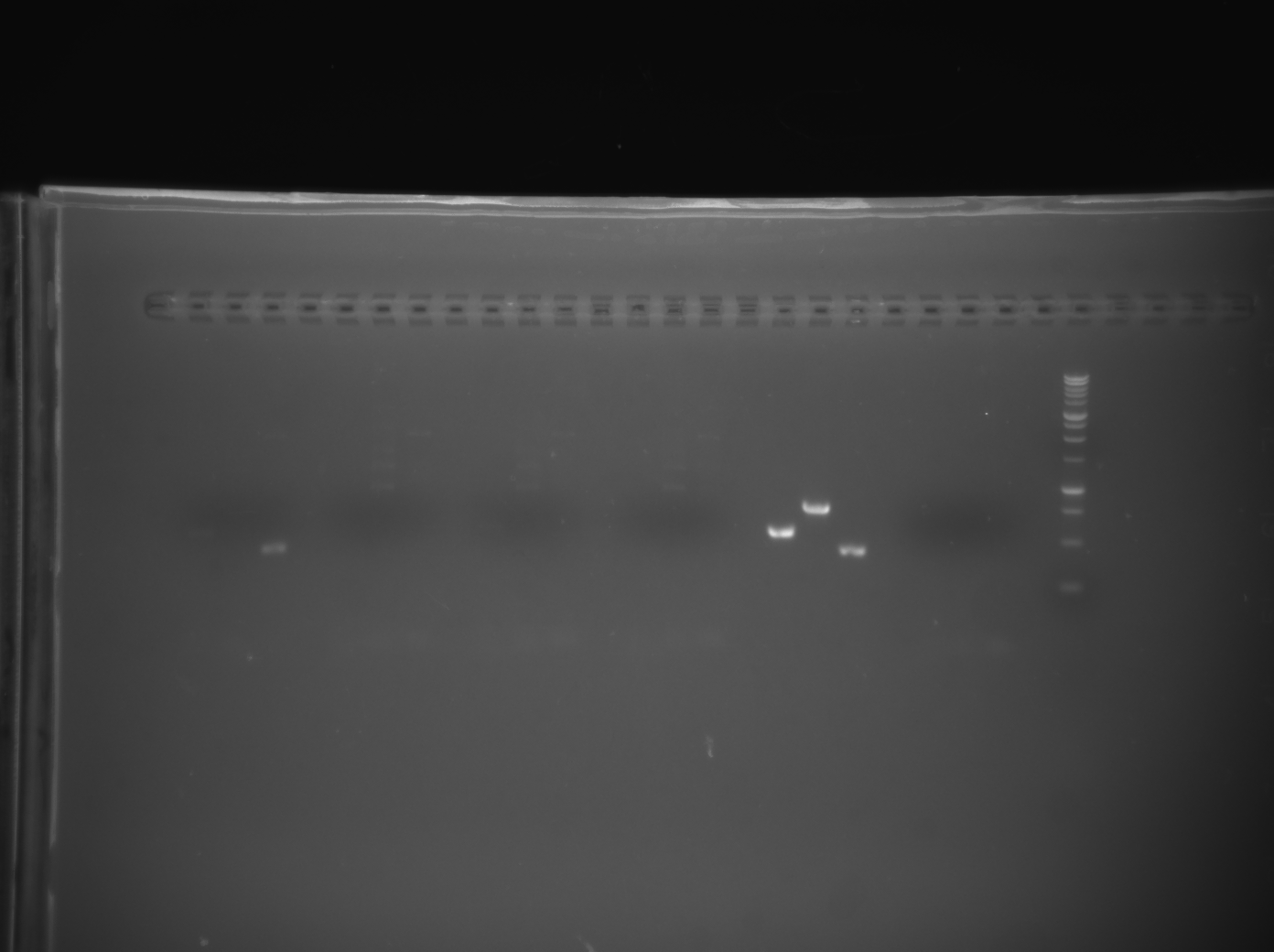

Supplement: Supplementary file 5 — Source Data Fig. 4 [file 44321_2024_57_MOESM5_ESM.zip › Fig 4/4D.tif]

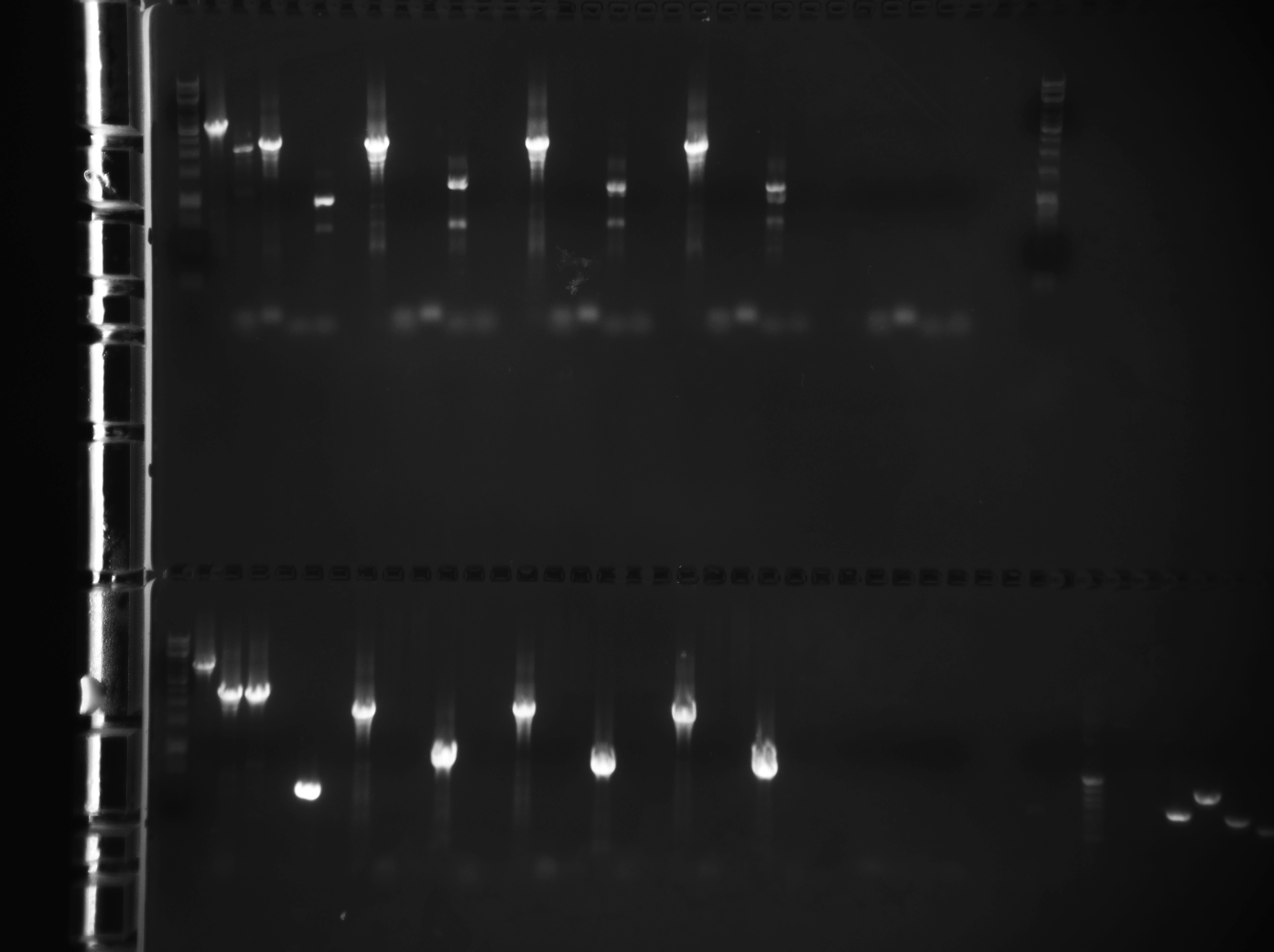

Supplement: Supplementary file 5 — Source Data Fig. 4 [file 44321_2024_57_MOESM5_ESM.zip › Fig 4/4B and 4C.tif]

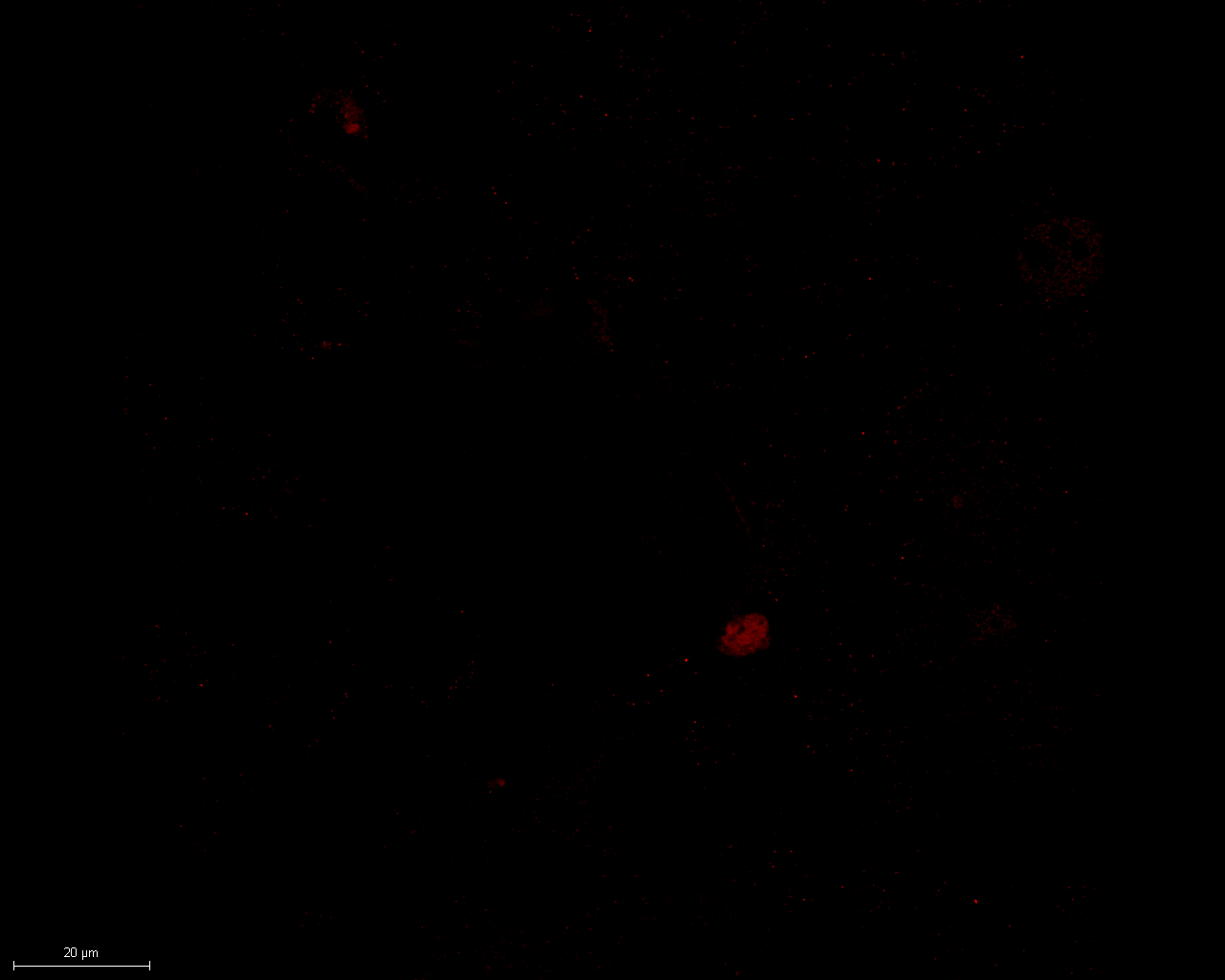

Supplement: Supplementary file 7 — Source Data Fig. 6 [file 44321_2024_57_MOESM7_ESM.zip › Fig 6/6F/larc2d7_mtip.tif]

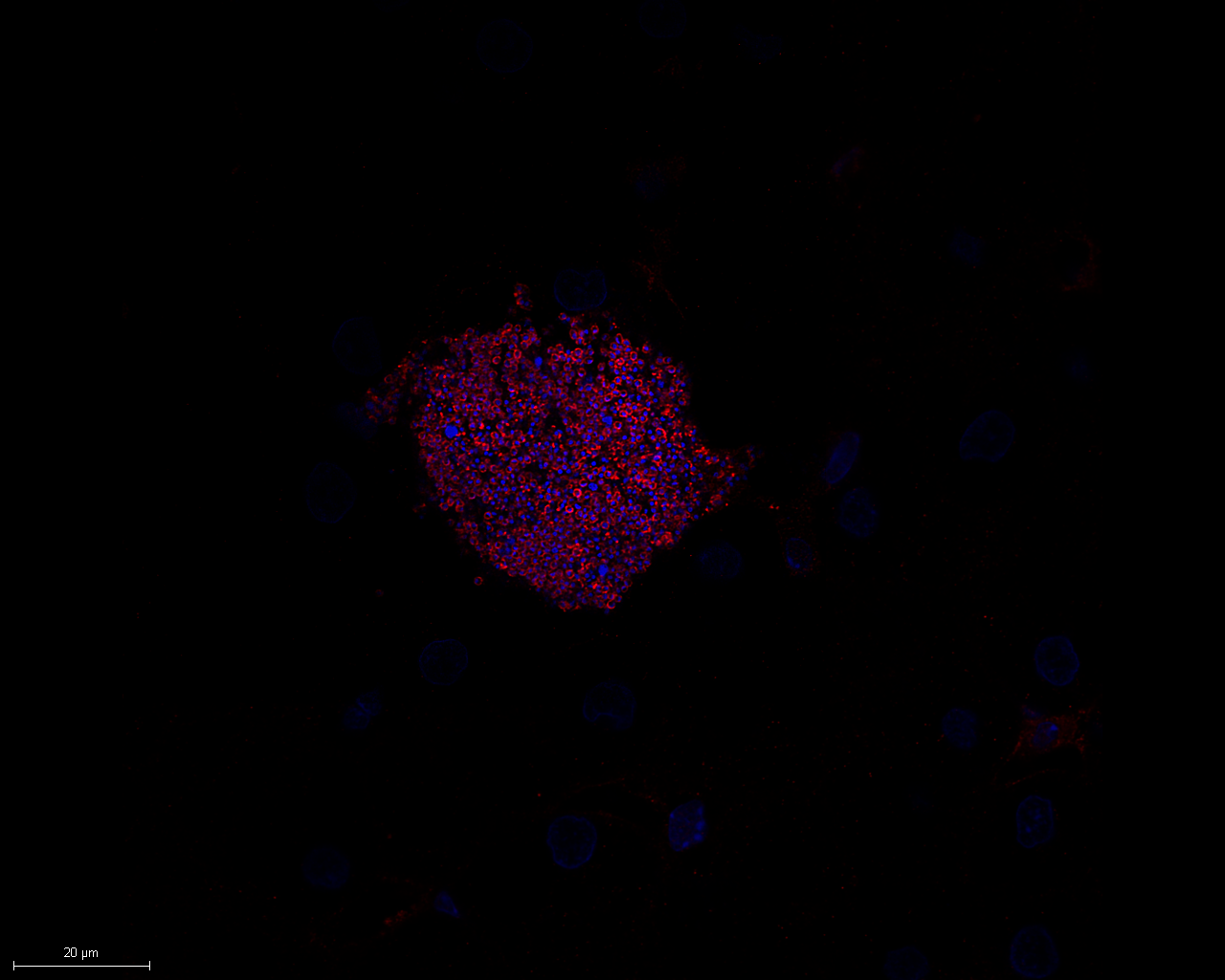

Supplement: Supplementary file 7 — Source Data Fig. 6 [file 44321_2024_57_MOESM7_ESM.zip › Fig 6/6F/wtd7_merge.tif]

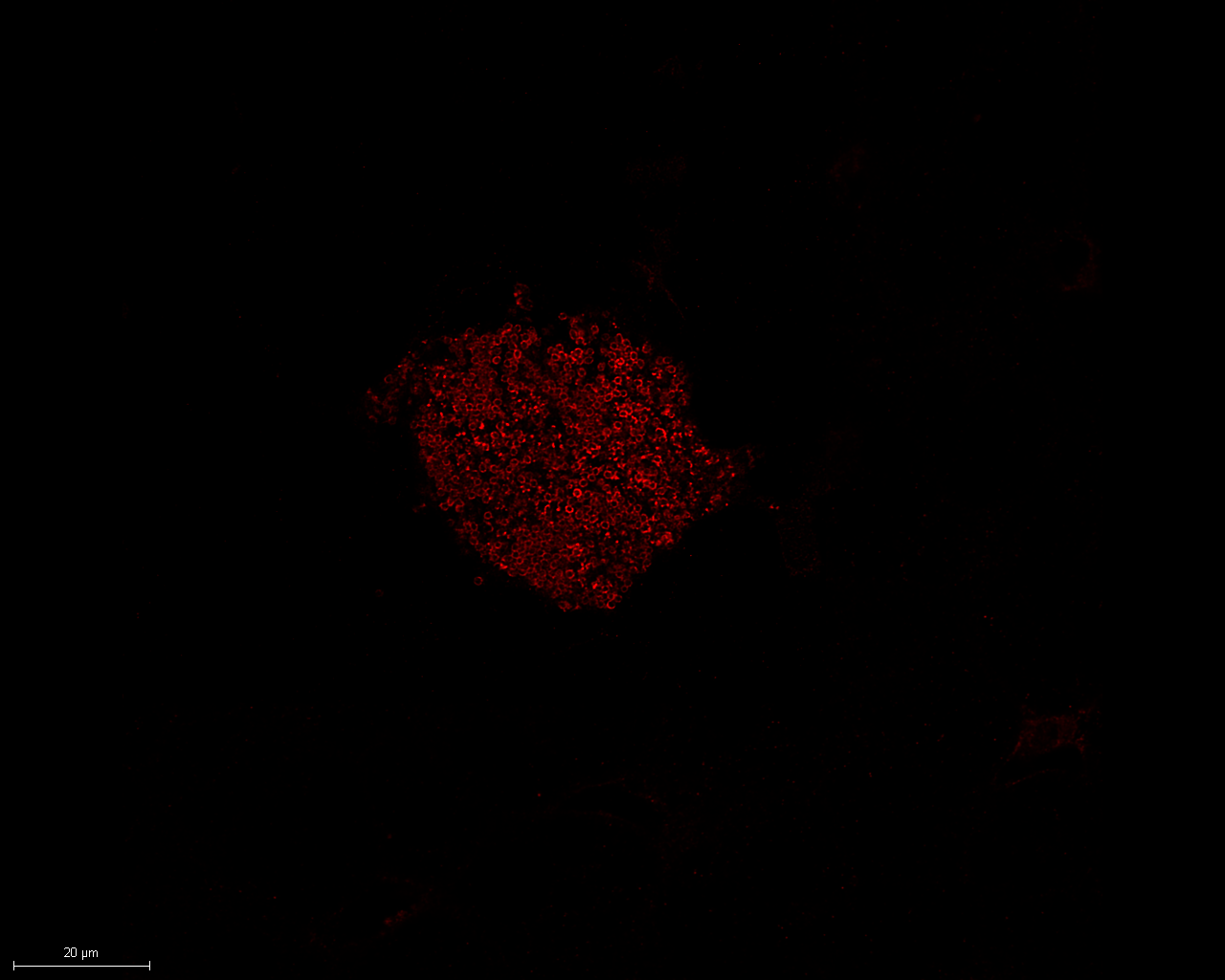

Supplement: Supplementary file 7 — Source Data Fig. 6 [file 44321_2024_57_MOESM7_ESM.zip › Fig 6/6F/wtd7_MTIP.tif]

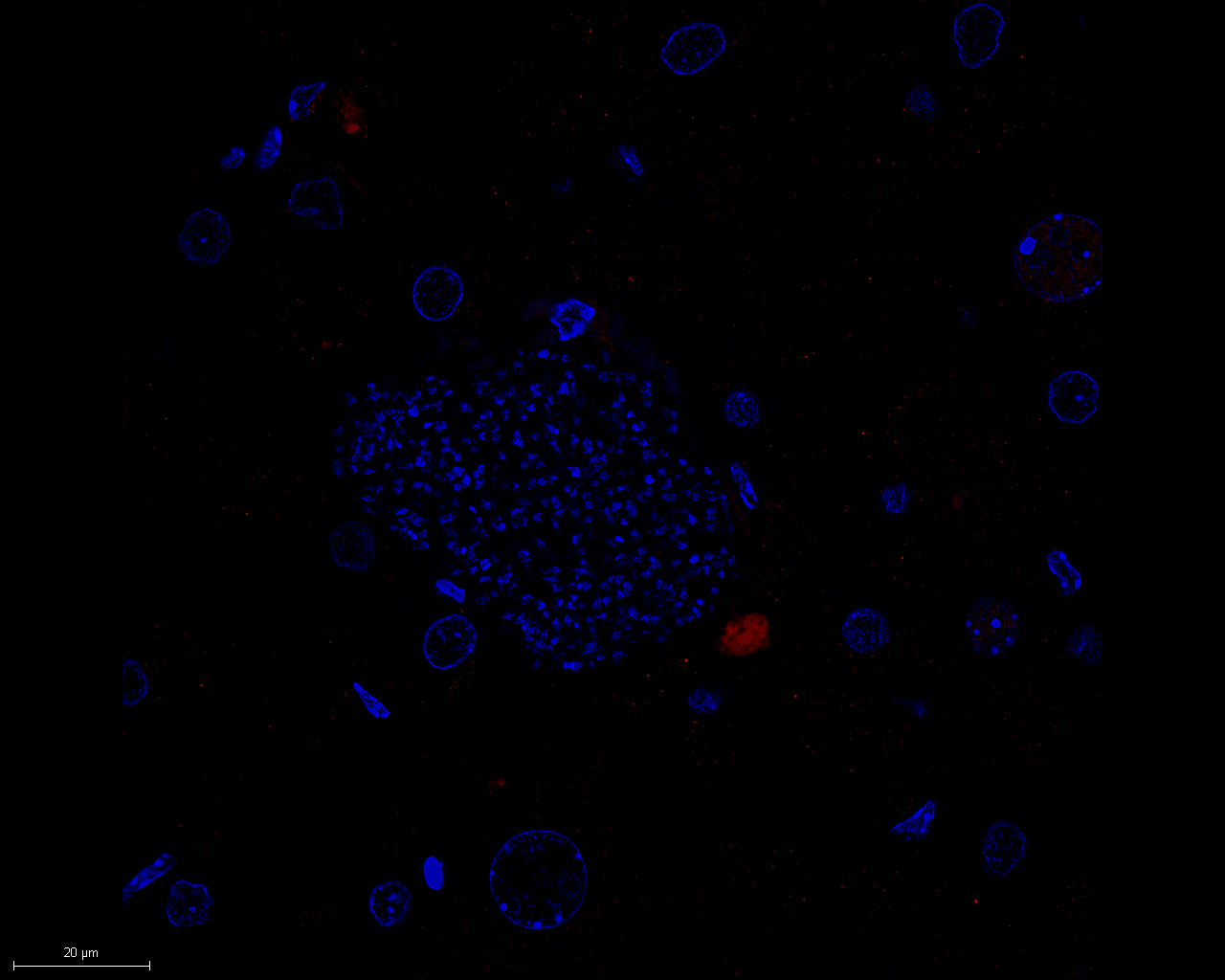

Supplement: Supplementary file 7 — Source Data Fig. 6 [file 44321_2024_57_MOESM7_ESM.zip › Fig 6/6F/larc2d7_merge_2.tif]

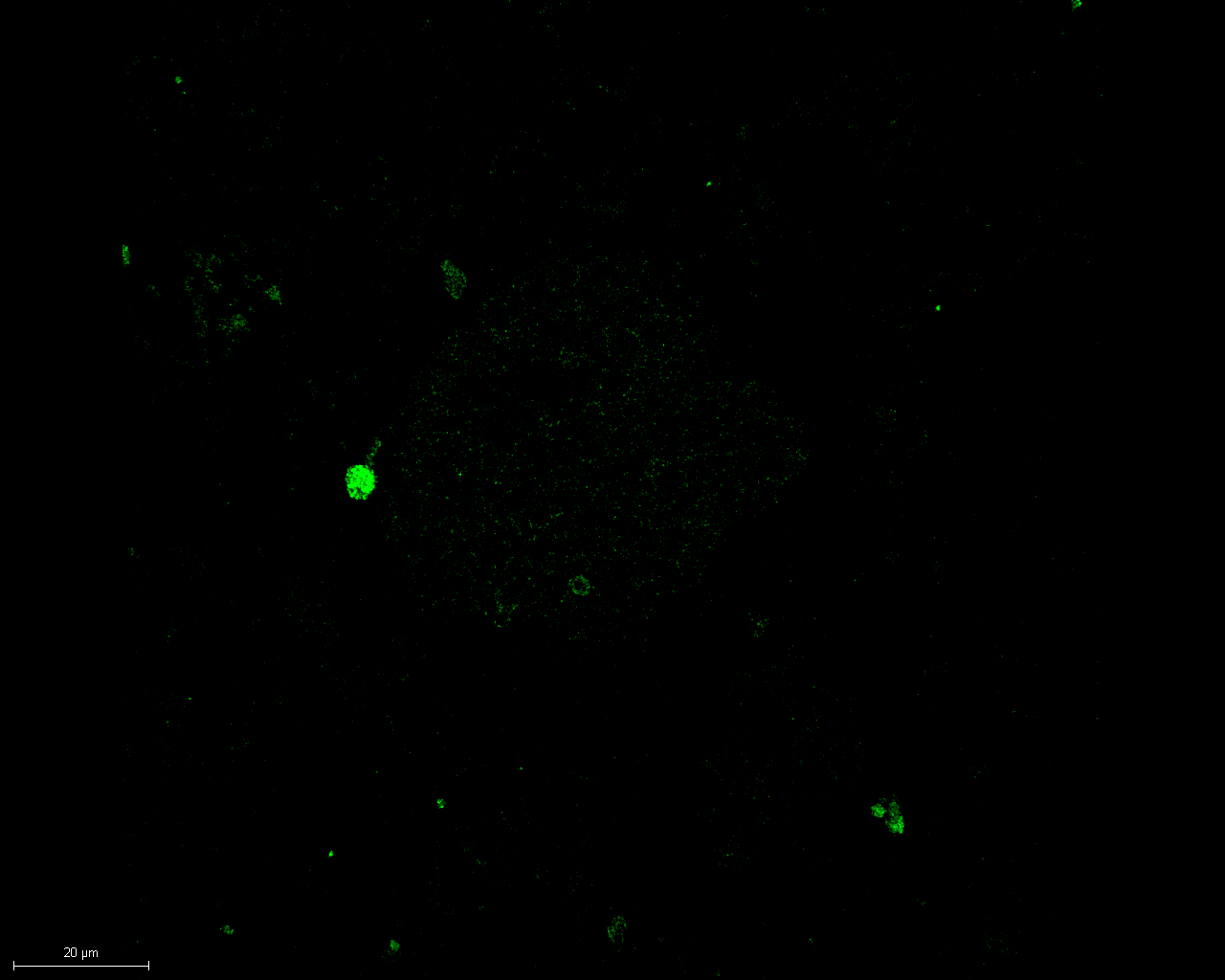

Supplement: Supplementary file 7 — Source Data Fig. 6 [file 44321_2024_57_MOESM7_ESM.zip › Fig 6/6E/larc2d7_msp1_1.tif]

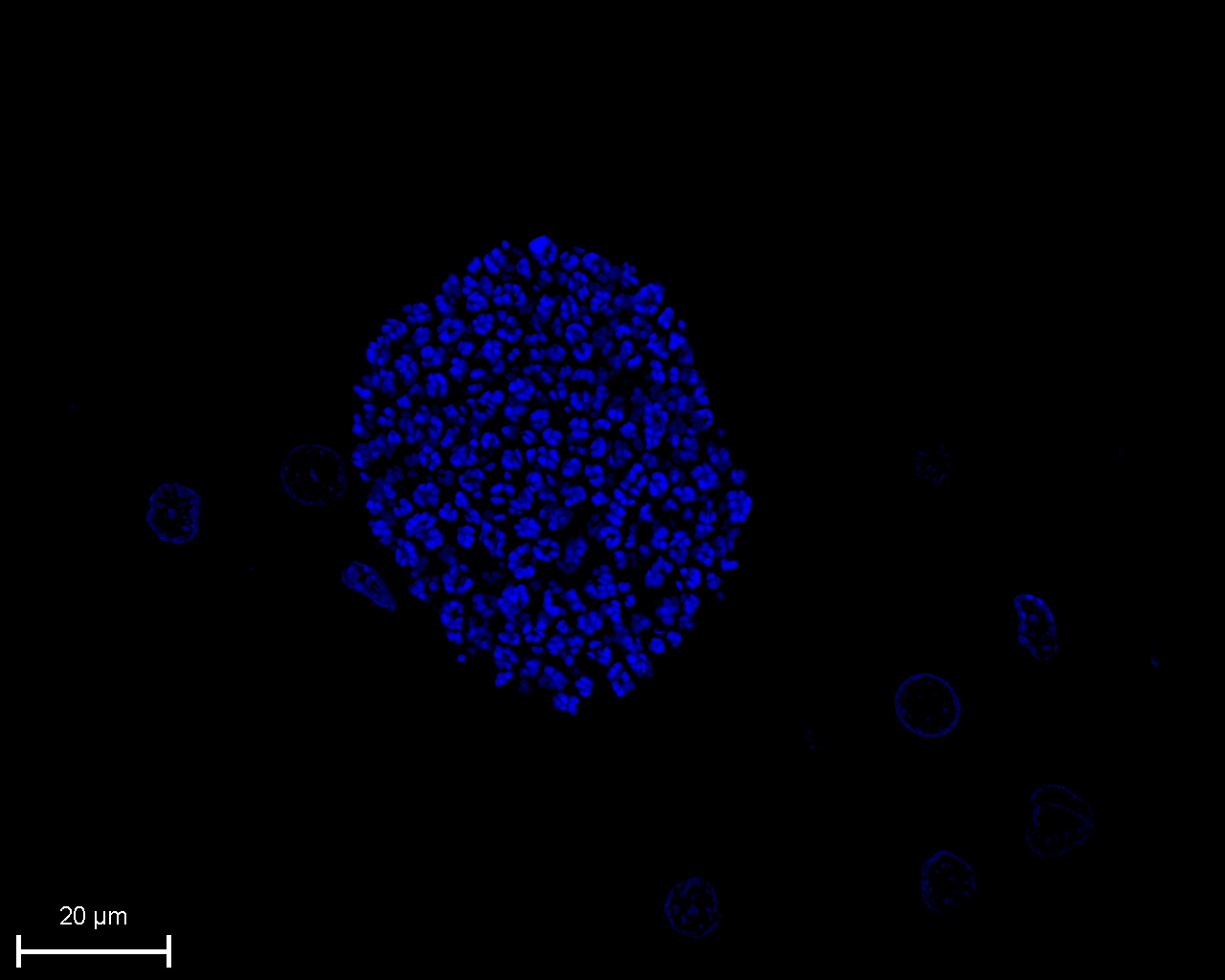

Supplement: Supplementary file 7 — Source Data Fig. 6 [file 44321_2024_57_MOESM7_ESM.zip › Fig 6/6E/wtd7_MSP1DAPI_3.tif]

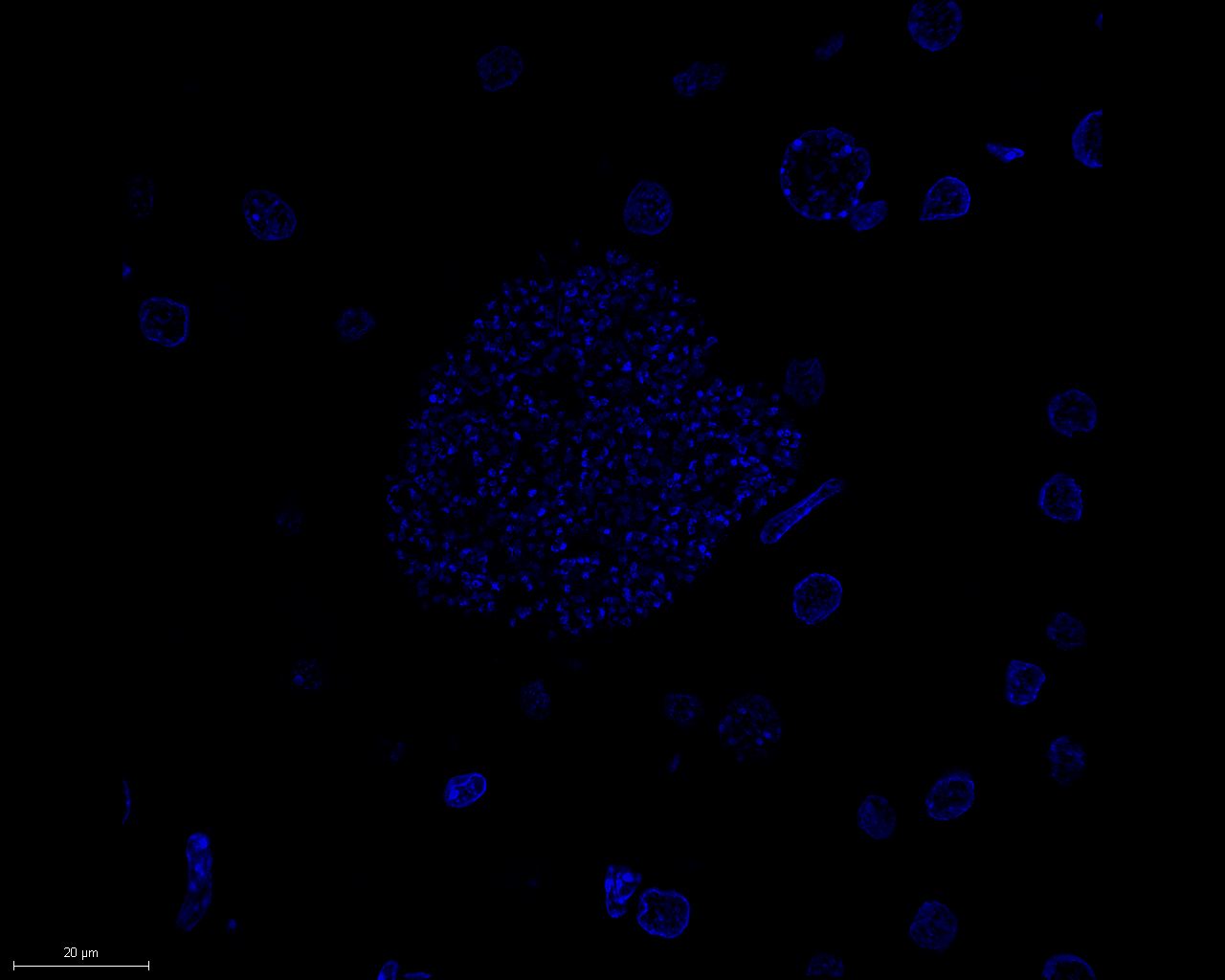

Supplement: Supplementary file 7 — Source Data Fig. 6 [file 44321_2024_57_MOESM7_ESM.zip › Fig 6/6E/larc2d7_dapi_1.tif]

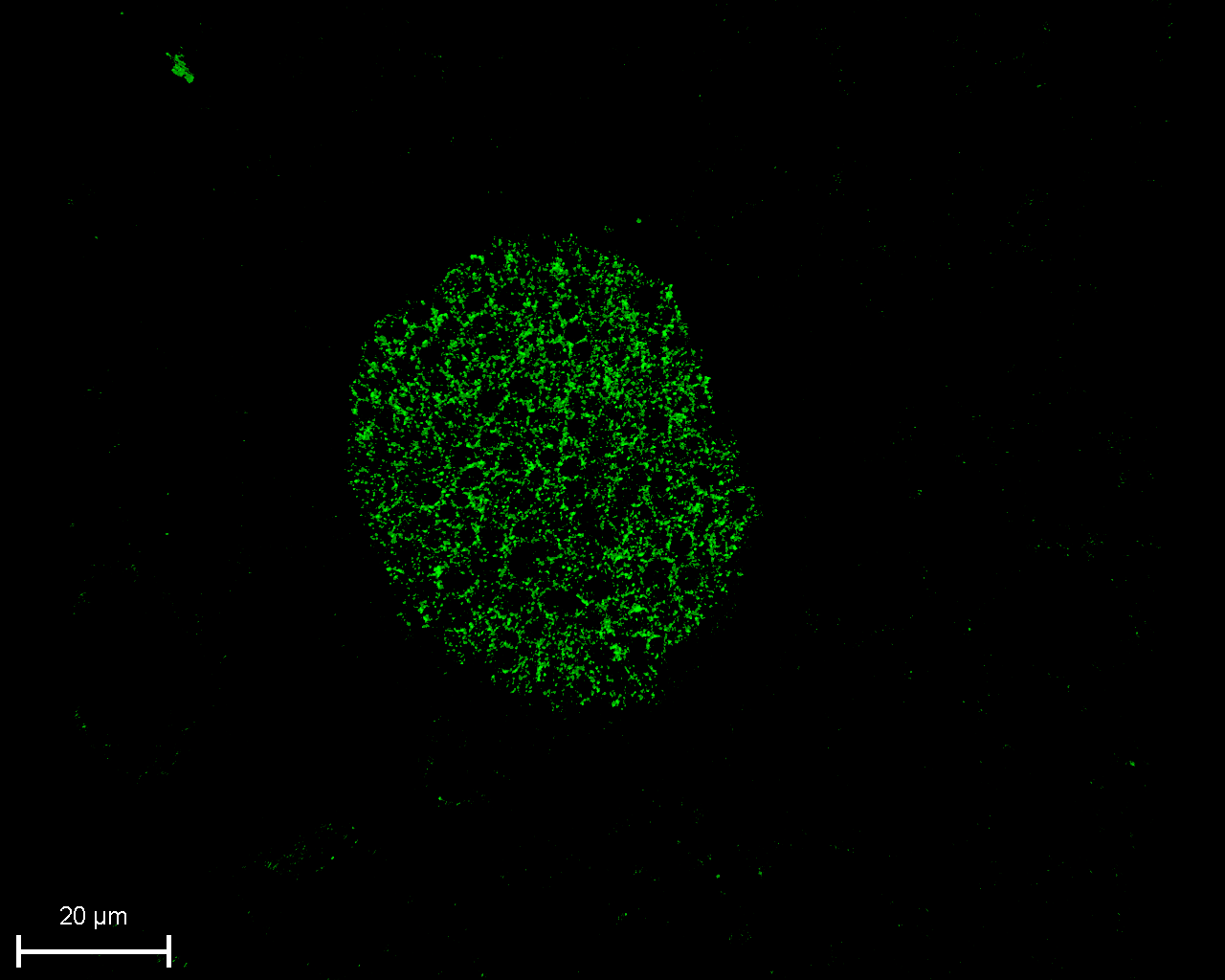

Supplement: Supplementary file 7 — Source Data Fig. 6 [file 44321_2024_57_MOESM7_ESM.zip › Fig 6/6E/wtd7_MSP1_3.tif]

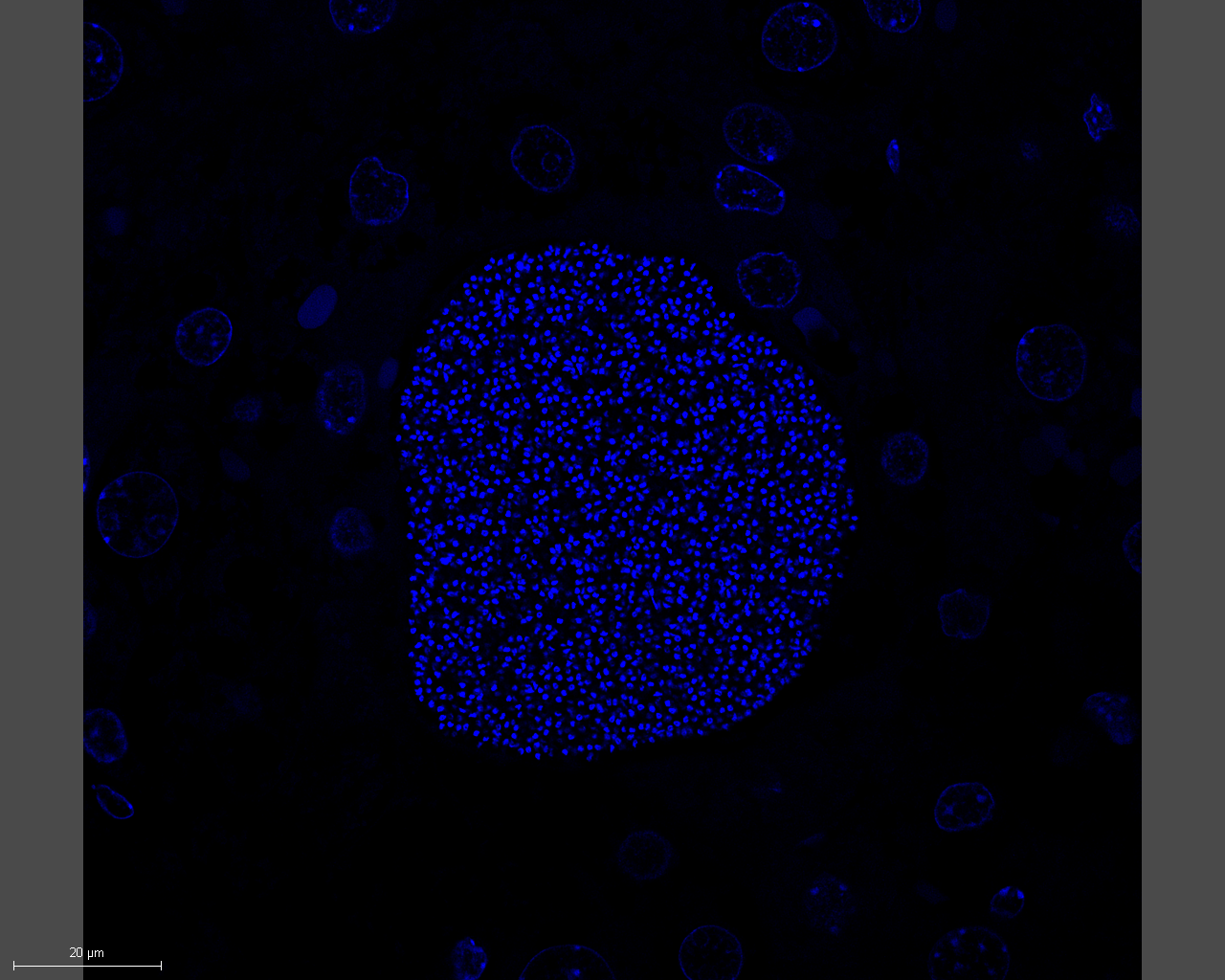

Supplement: Supplementary file 7 — Source Data Fig. 6 [file 44321_2024_57_MOESM7_ESM.zip › Fig 6/6D/wtd7_cspExp1_PVM_dapi.tif]

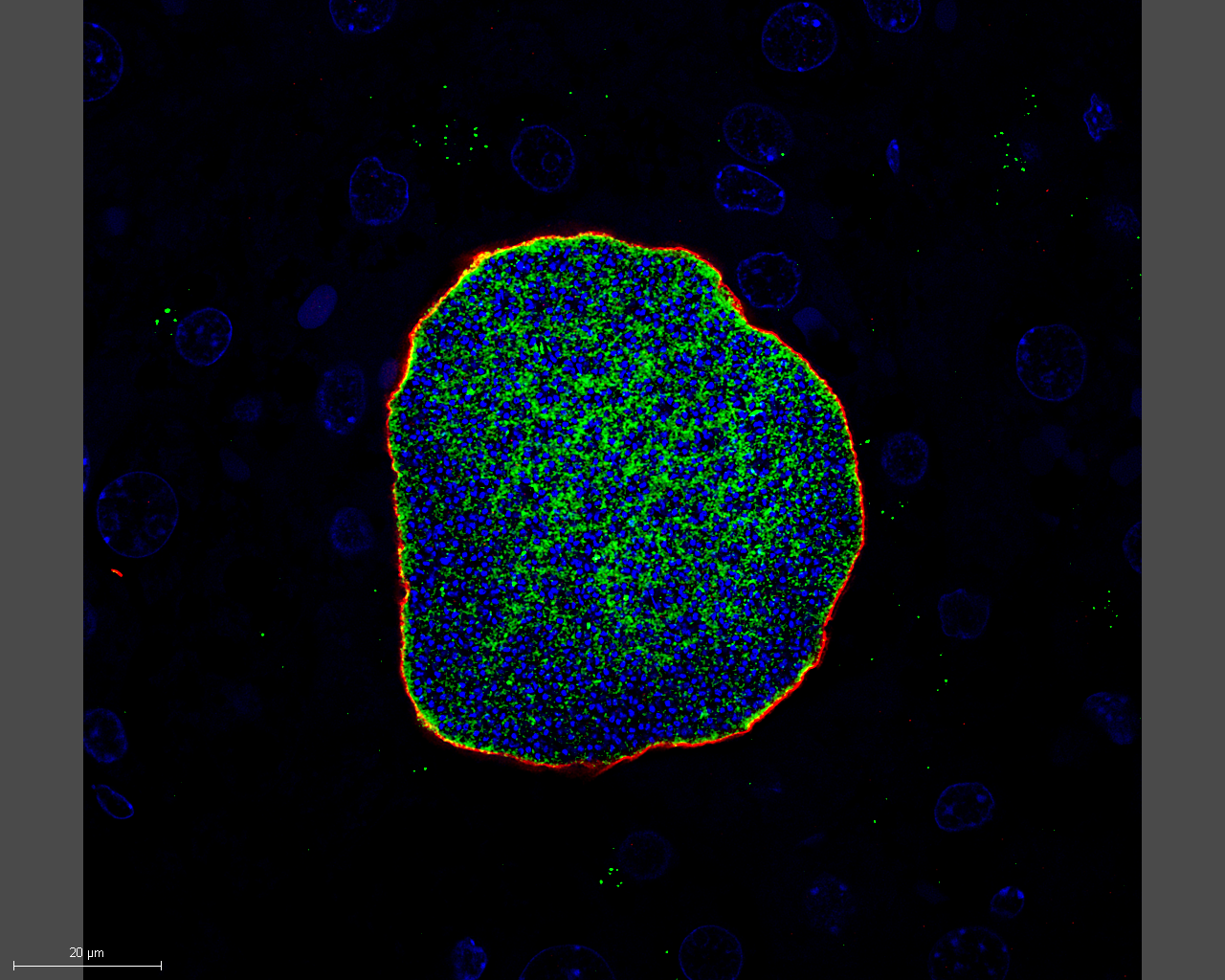

Supplement: Supplementary file 7 — Source Data Fig. 6 [file 44321_2024_57_MOESM7_ESM.zip › Fig 6/6D/wtd7_cspExp1_PVM_Merge.tif]

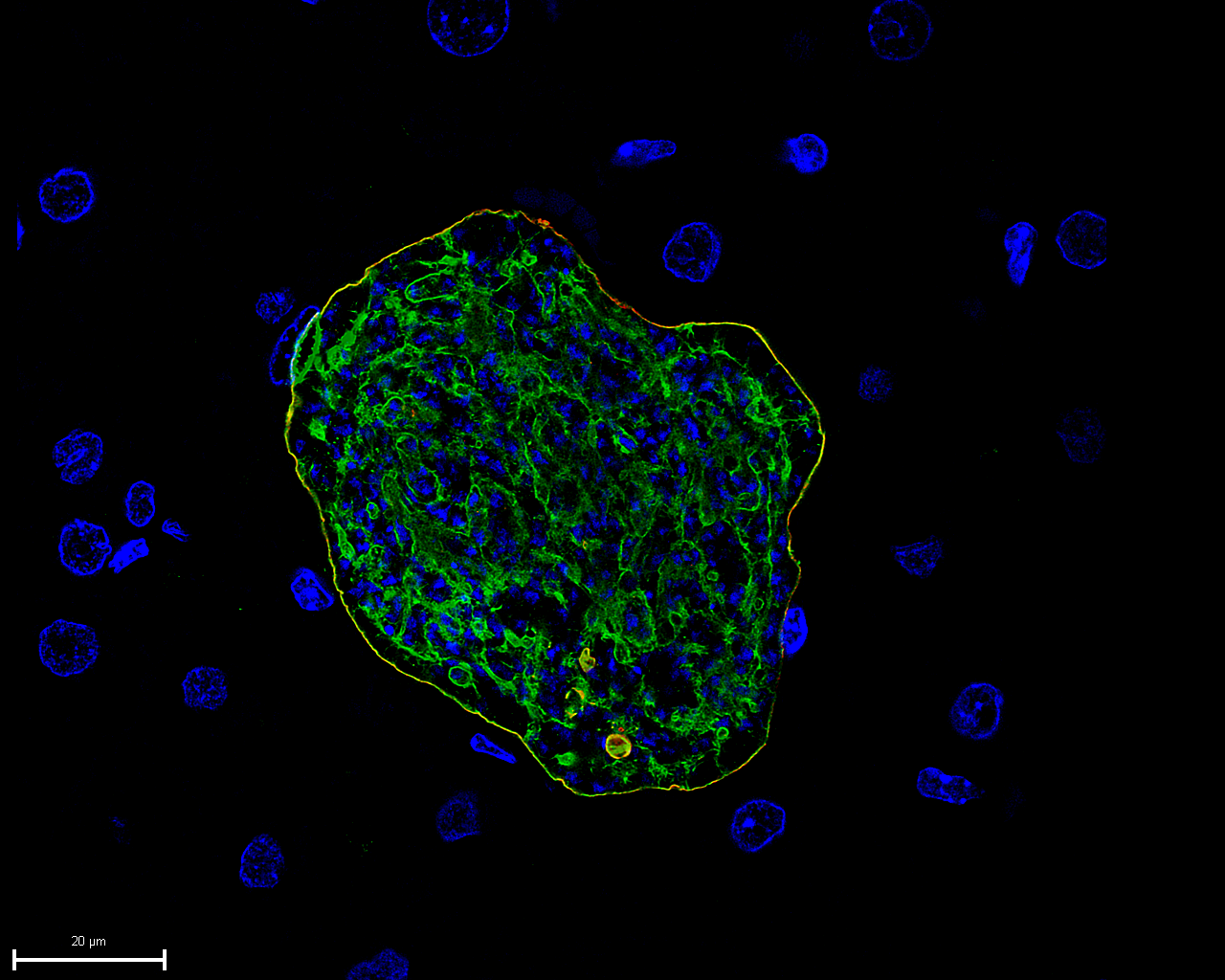

Supplement: Supplementary file 7 — Source Data Fig. 6 [file 44321_2024_57_MOESM7_ESM.zip › Fig 6/6D/LARC2D7_merge.tif]

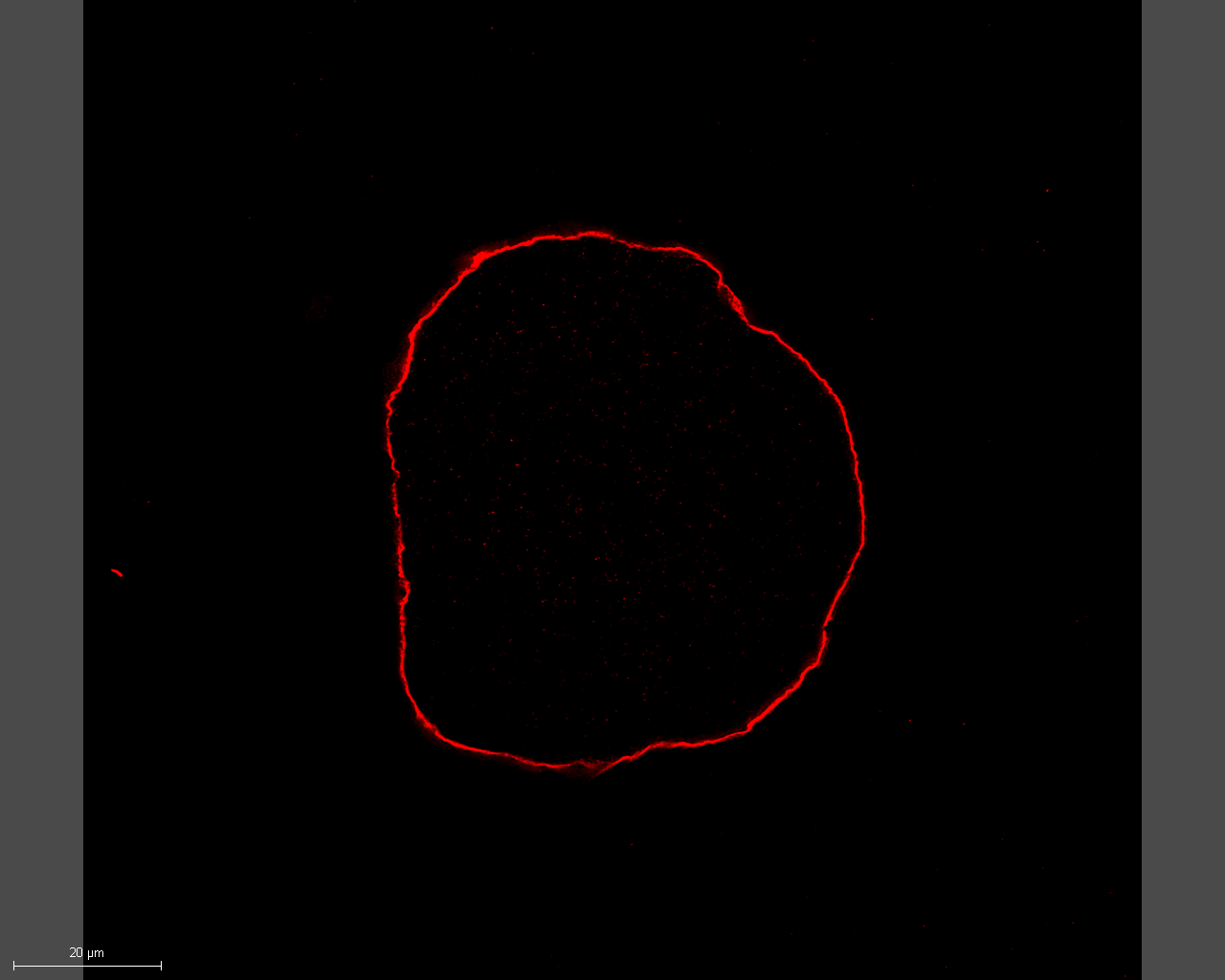

Supplement: Supplementary file 7 — Source Data Fig. 6 [file 44321_2024_57_MOESM7_ESM.zip › Fig 6/6D/wtd7_cspExp1_PVM_Exp1.tif]

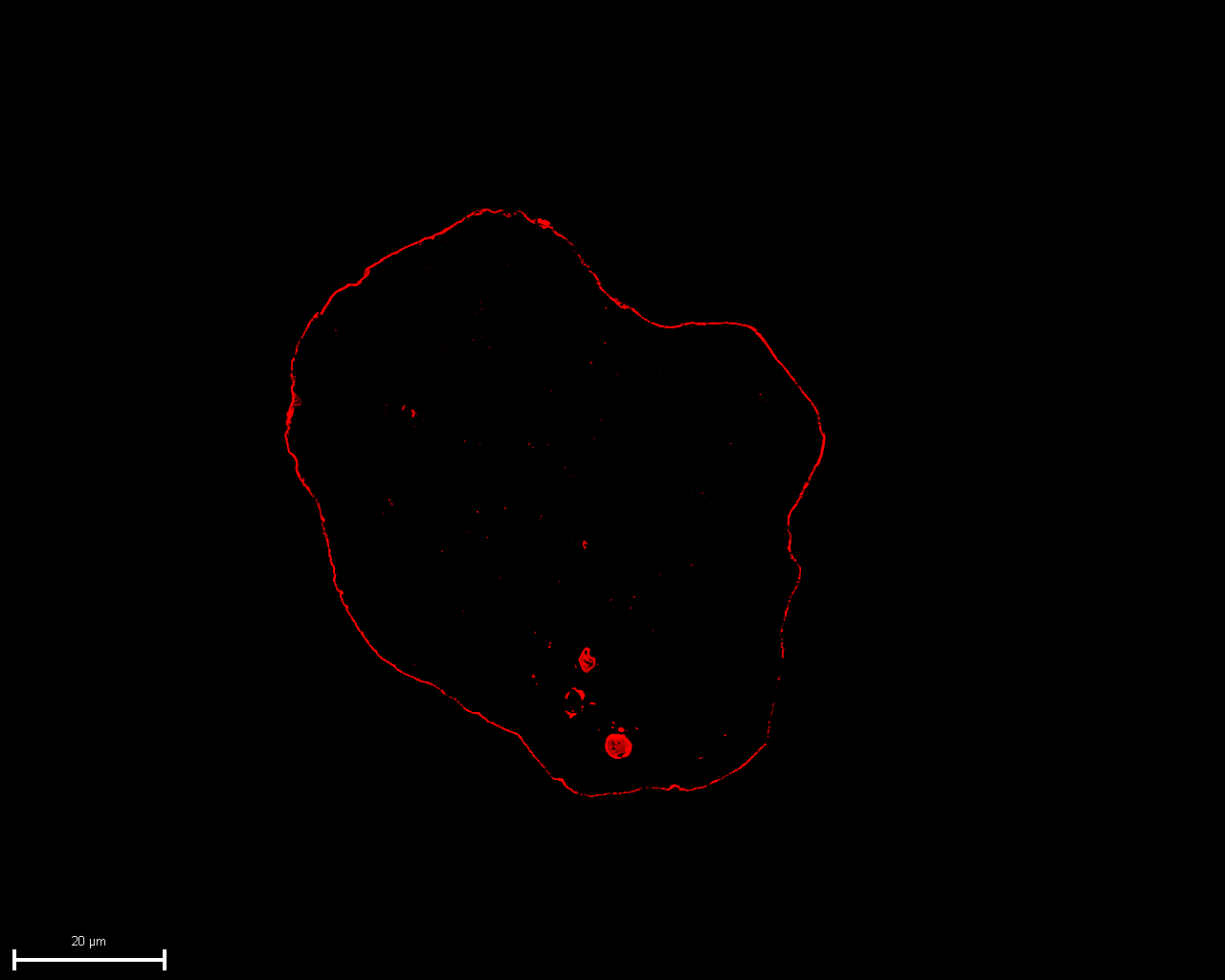

Supplement: Supplementary file 7 — Source Data Fig. 6 [file 44321_2024_57_MOESM7_ESM.zip › Fig 6/6D/LARC2D7_Exp1.tif]

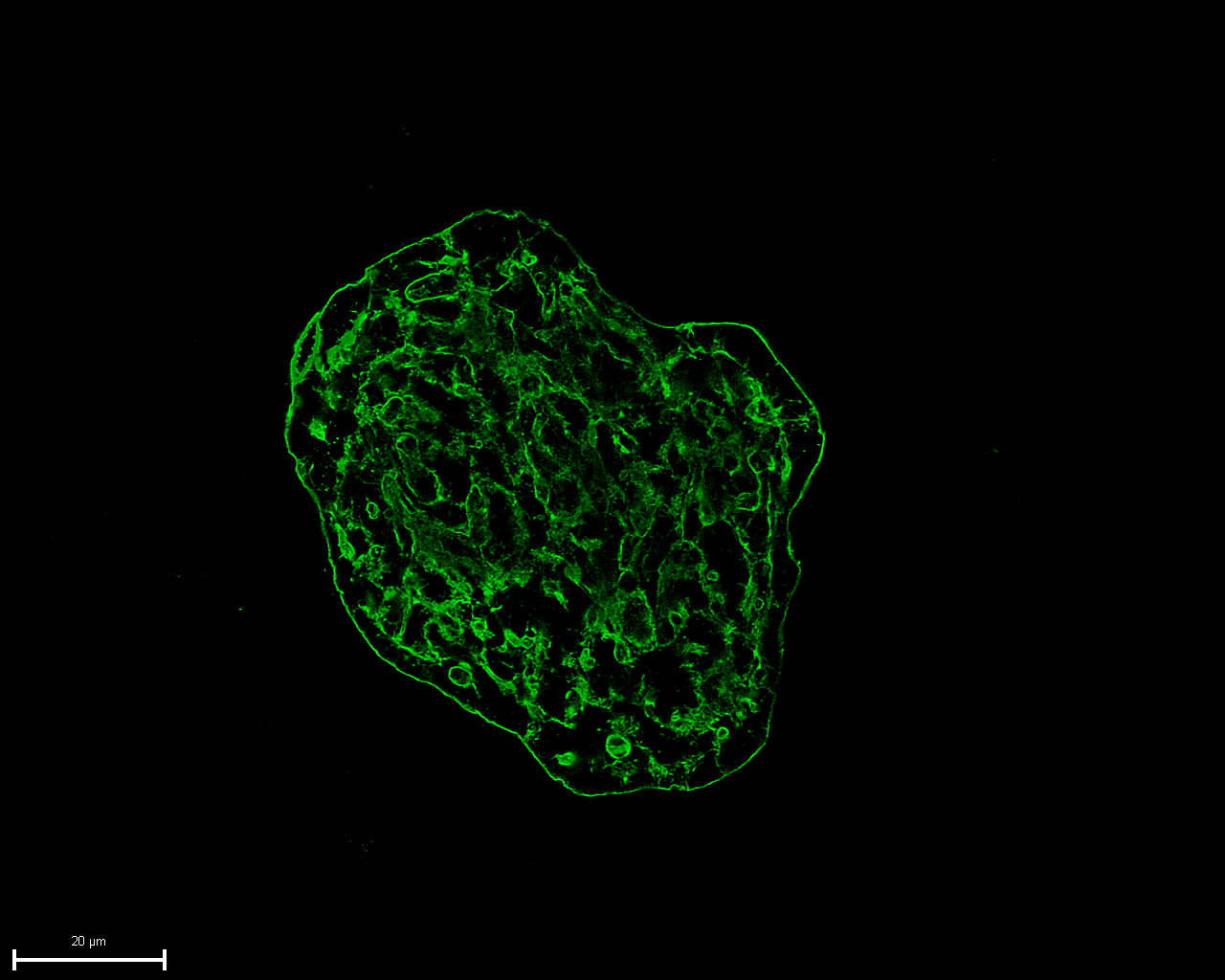

Supplement: Supplementary file 7 — Source Data Fig. 6 [file 44321_2024_57_MOESM7_ESM.zip › Fig 6/6D/LARC2D7_CSP.tif]

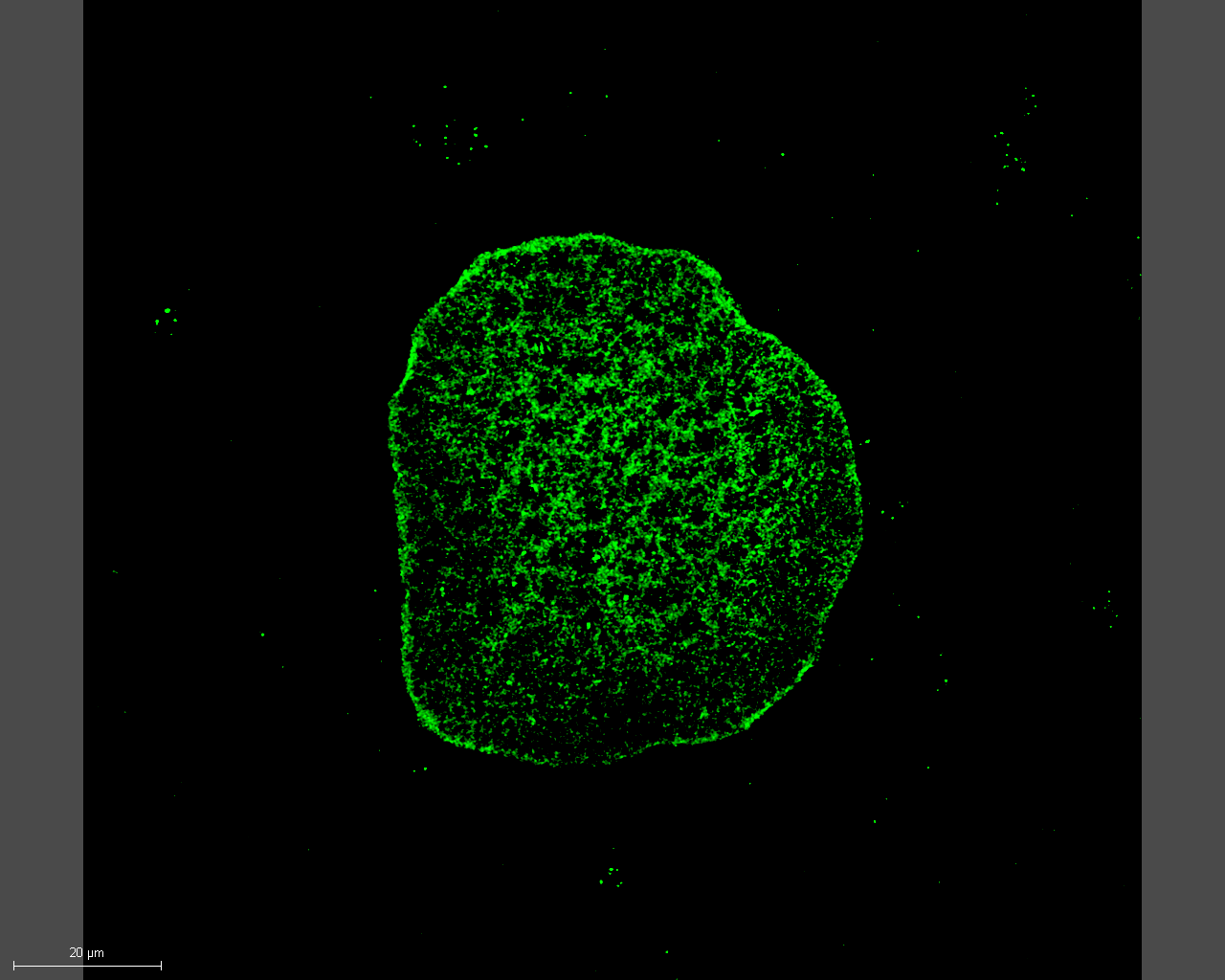

Supplement: Supplementary file 7 — Source Data Fig. 6 [file 44321_2024_57_MOESM7_ESM.zip › Fig 6/6D/wtd7_cspExp1_PVM_csp.tif]

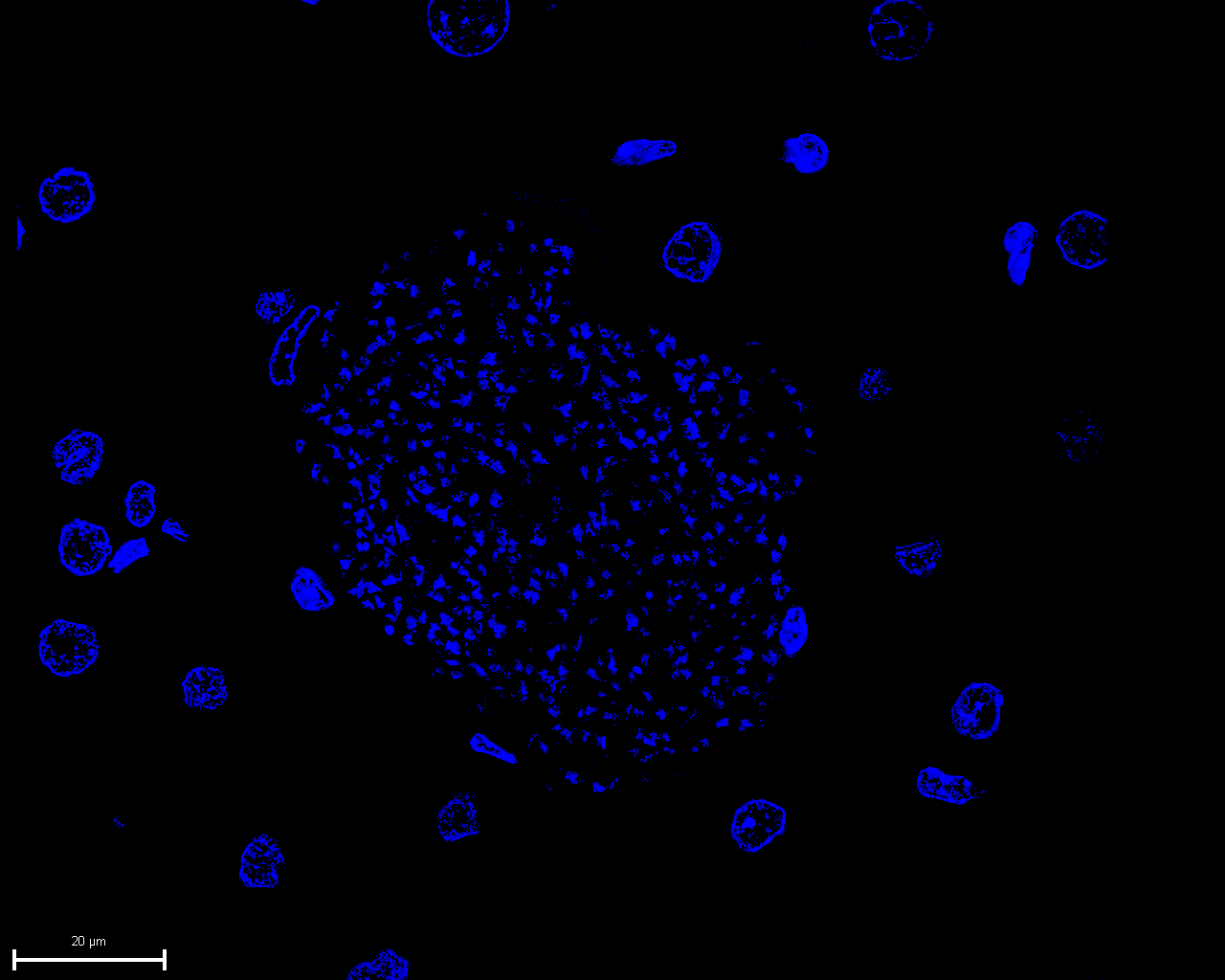

Supplement: Supplementary file 7 — Source Data Fig. 6 [file 44321_2024_57_MOESM7_ESM.zip › Fig 6/6D/LARC2D7_dapi.tif]
