# Supplementary material for: A replication competent Plasmodium falciparum parasite completely attenuated by dual gene deletion
Source: EMBO Mol Med. 2024 Mar 21;16(4):10. doi: 10.1038/s44321-024-00057-7 (PMC11018819; doi:10.1038/s44321-024-00057-7)
Supplement: Supplementary file 10 — Expanded View Figures [file 44321_2024_57_MOESM10_ESM.pdf]

Expanded View Figures

Figure EV1. PyLARC2 immunization elicits long-lived CD8 + T cell-based immunity, especially liver resident CD8 + T cells.

(**A**) Experimental schematics for (**B–E**). Nine C57BL/6 mice were immunized three times with 50,000 PyLARC2 sporozoites 1 month apart. Five mock immunized mice received mosquito debris from uninfected salivary glands as a control. Mice were euthanized at 2 months after the last immunization and the livers and spleens were collected for T cell analysis via flow cytometry. (**B**) (i) frequencies and (ii) the total number of antigen experienced $CD8^+$ memory T cells ($CD44^{hi}$) in the livers of mock or PyLARC2 immunized mice. (**C**) (i) frequencies, (ii) total number, and (iii) representative flow layout of $CD69^{hi}CXCR6^{hi}$liver-resident memory $CD8^+$ T cells (T_{RM}) (gated on $CD44^{hi}CD8\alpha^+$ singlet events). (**D**) Frequencies and a representative flow layout of $CD11a^{hi}CD44^{hi}$ $CD8^+$ T cells in the liver. (**E**) (i) frequencies, (ii) total number, and (iii) representative flow layout of $CD44^{hi}CD62L^{lo}$ $CD8^+$ effector memory T cells (T_{EM}) in the spleen. Data is shown as mean ± SD. Statistical analysis was done using non-parametric Mann–Whitney test. *P* values are indicated in the figure. $P > 0.05$ is taken as not significant.

© The Author(s)

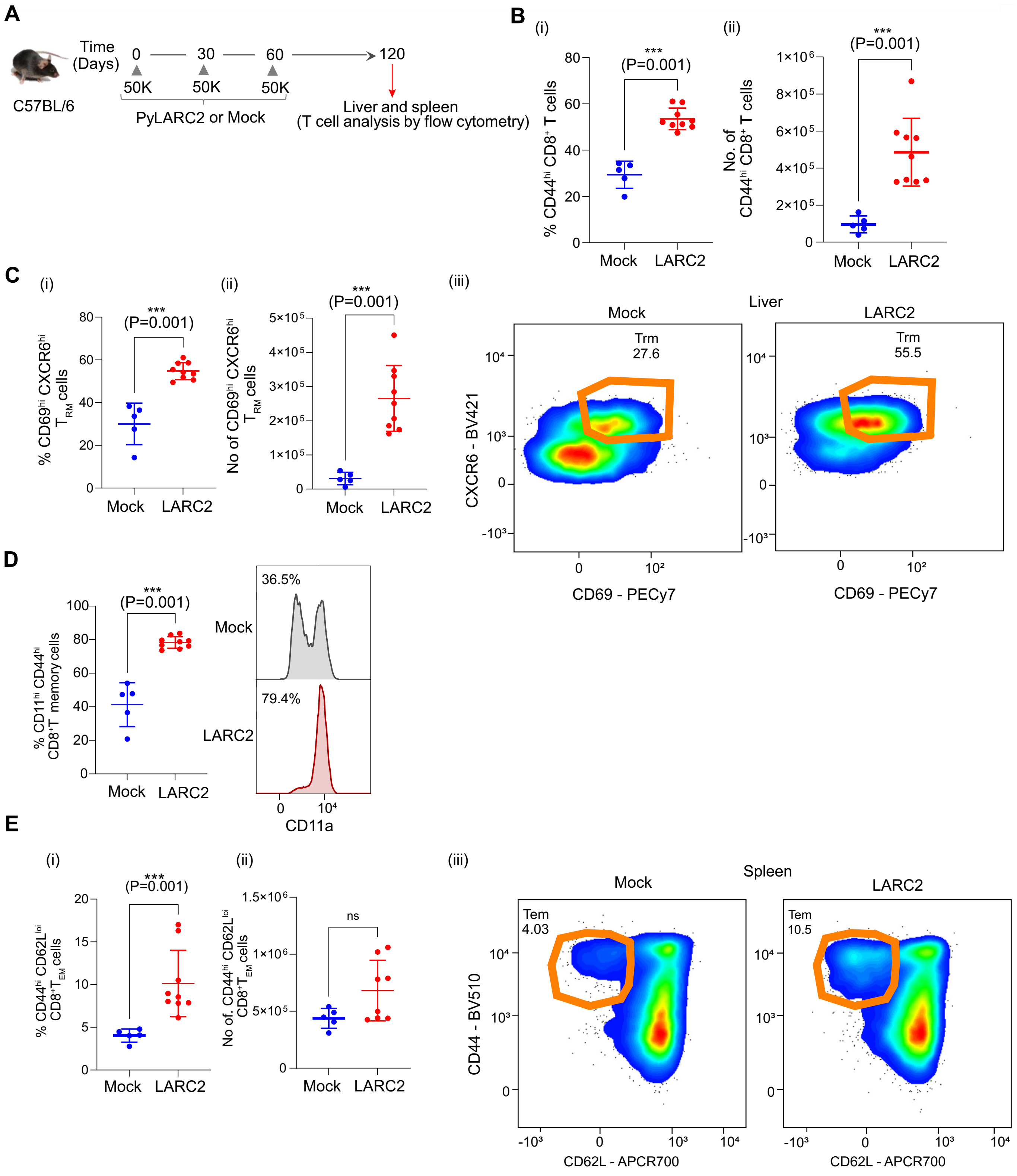
A
Time (Days)
0
30
60
120
50K
50K
50K
C57BL/6
PyLARC2 or Mock
Liver and spleen
(T cell analysis by flow cytometry)
B
(i)
(ii)
% CD44hi CD8+ T cells
No. of CD44hi CD8+ T cells
*** (P=0.001)
Mock
LARC2
C
(i)
(ii)
(iii)
% CD69hi CXCR6hi TRM cells
No of CD69hi CXCR6hi TRM cells
Liver
Mock
LARC2
Trm 27.6
Trm 55.5
CXCR6 - BV421
CD69 - PECy7
D
% CD11hi CD44hi CD8+T memory cells
36.5%
79.4%
Mock
LARC2
CD11a
E
(i)
(ii)
(iii)
% CD44hi CD62Llo CD8+TEM cells
No of. CD44hi CD62Llo CD8+TEM cells
ns
Spleen
Tem 4.03
Tem 10.5
CD44 - BV510
CD62L - APCR700

 © The Author(s)

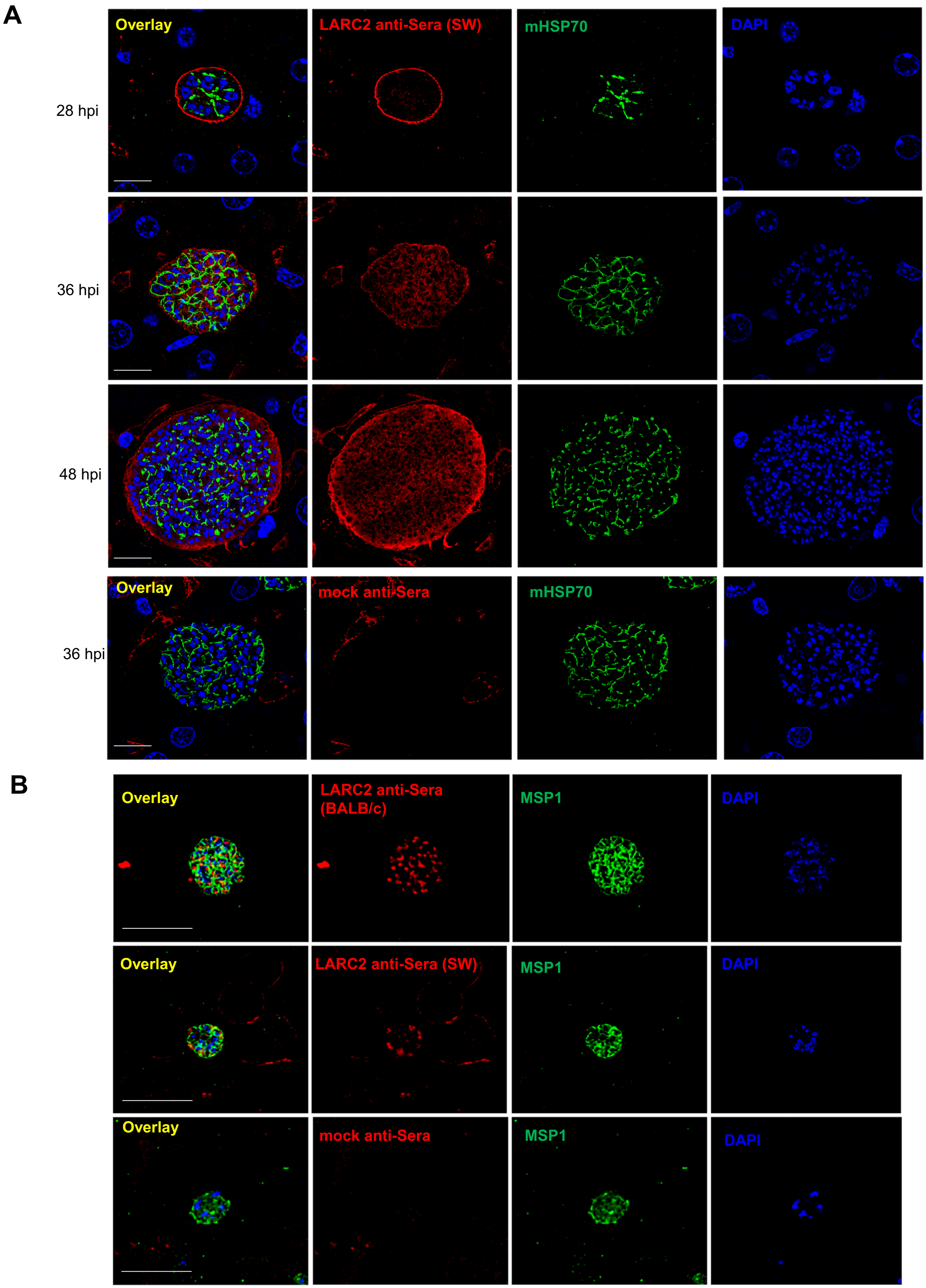
A
Overlay
LARC2 anti-Sera (SW)
mHSP70
DAPI
28 hpi
36 hpi
48 hpi
Overlay
mock anti-Sera
mHSP70
36 hpi
B
Overlay
LARC2 anti-Sera (BALB/c)
MSP1
DAPI
Overlay
LARC2 anti-Sera (SW)
MSP1
DAPI
Overlay
mock anti-Sera
MSP1
DAPI

◀ **Figure EV2. PyLARC2 immunization of elicits humoral responses to liver and blood stages.**

(**A**) Plasma was collected from six PyLARC2 immunized SW mice and pooled. The pooled plasma was then diluted 1:100 and used to evaluate reactivity to antigens at different timepoints of PyWT LS development using IFA. Sera is shown in red, anti-mHSP70 antibody is used as control (green) and DNA is stained with DAPI (blue). Scale bar is 20 µm. At early timepoints of LS development, the immune sera from PyLARC2 immunized mice reacted with antigens expressed on the PVM or PPM. In more mature LS, both cytoplasmic and membrane reactivity was observed, indicating that the serum antibodies recognized antigens located in both the cytoplasm and the membranes of the parasite. No reactivity was observed in plasma harvested from mock-immunized mice, indicating that the staining was specific to the PyLARC2 immunized mice. (**B**) Plasma was collected from six PyLARC2 immunized SW mice or BALB/cJ mice. Pooled plasma for each mouse strain was diluted 1:100 and used to evaluate reactivity to antigens expressed in PyWT blood stages. Sera is shown in red, anti-MSP1antibody is used as control (green) and DNA is stained with DAPI (blue). Scale bar is 10 µm. Immune sera from both SW and BALB/cJ mice reacted with antigens expressed in the cytoplasm of blood stages. No reactivity was observed in plasma harvested from mock-immunized mice, indicating that the staining was specific to immune sera from PyLARC2 immunized mice.

 © The Author(s)

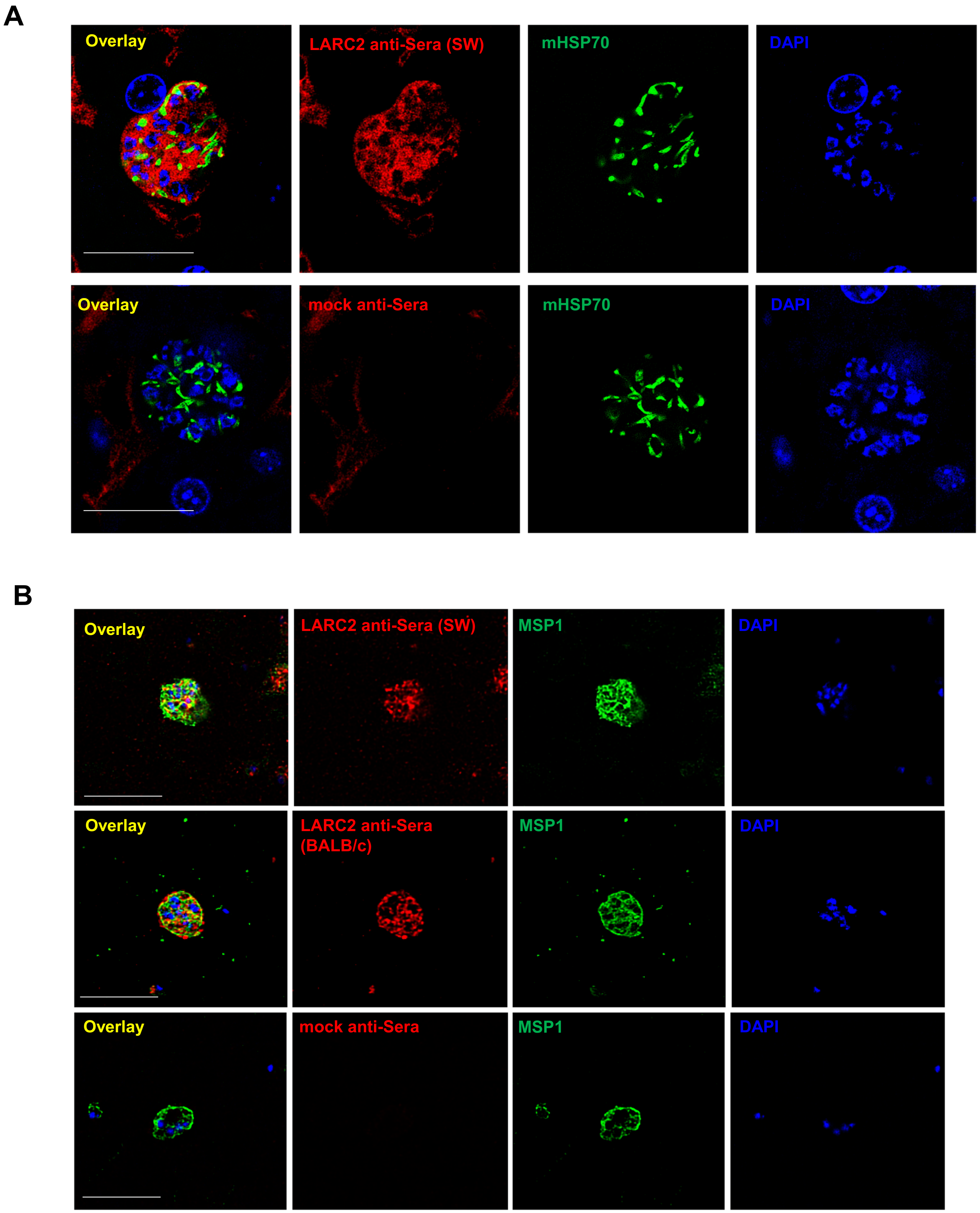
A
Overlay
LARC2 anti-Sera (SW)
mHSP70
DAPI
Overlay
mock anti-Sera
mHSP70
DAPI
B
Overlay
LARC2 anti-Sera (SW)
MSP1
DAPI
Overlay
LARC2 anti-Sera (BALB/c)
MSP1
DAPI
Overlay
mock anti-Sera
MSP1
DAPI

◀ **Figure EV3. PyLARC2 immunization of elicits cross-species humoral responses against *P. berghei* liver and blood stages.**

(**A**) Plasma was collected from six PyLARC2 immunized SW mice and pooled. The pooled plasma was then diluted 1:100 and used to evaluate reactivity of immune sera to antigens at different timepoints of *P. berghei* ANKA WT LS development at 36 hpi using IFA. Sera is shown in red, anti-mHSP70 antibody is used as control (green) and DNA is stained with DAPI (blue). Scale bar is 20 µm. In more mature *P. berghei* LS, PyLARC2 IgG displayed cross-reactivity to *P. berghei* antigens located in both the cytoplasm and the membranes of the parasite. No reactivity was observed in plasma harvested from mock-immunized mice, indicating that the staining was specific to the PyLARC2 immunized mice. (**B**) Plasma was collected from six PyLARC2 immunized SW or BALB/cJ mice. Pooled plasma for each mouse strain was diluted 1:100 and used to evaluate reactivity of immune sera to antigens expressed in *P. berghei* ANKA WT blood stages. Sera is shown in red, anti-MSP1antibody is used as control (green) and DNA is stained with DAPI (blue). Scale bar is 10 µm. Immune sera from both SW and BALB/cJ mice reacted with antigens expressed in the cytoplasm of blood stages. No reactivity was observed in plasma harvested from mock-immunized mice, indicating that the staining was specific to immune sera from PyLARC2 immunized mice.

© The Author(s)

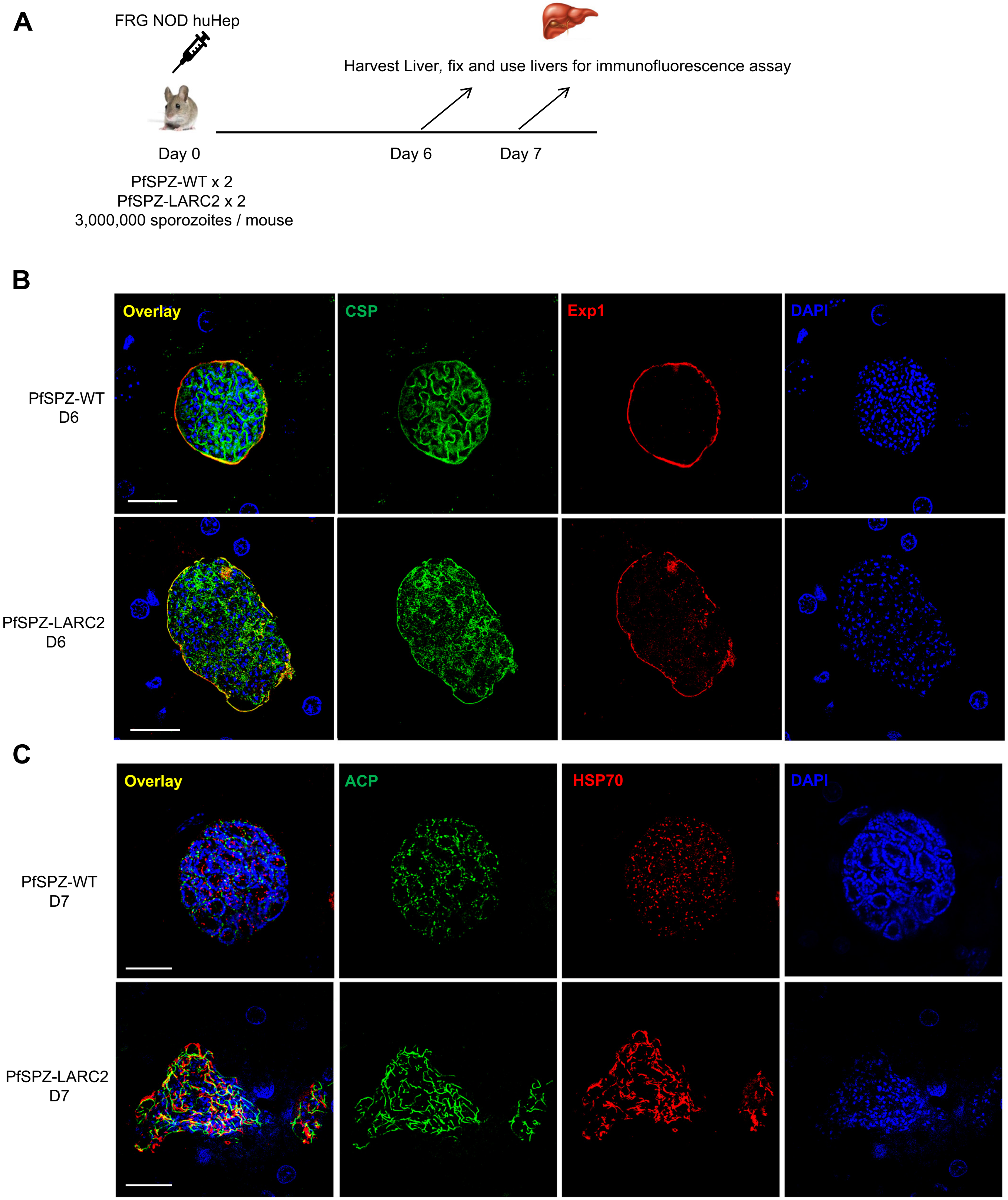
A
FRG NOD huHep
Harvest Liver, fix and use livers for immunofluorescence assay
Day 0
Day 6
Day 7
PfSPZ-WT x 2
PfSPZ-LARC2 x 2
3,000,000 sporozoites / mouse
B
Overlay
CSP
Exp1
DAPI
PfSPZ-WT D6
PfSPZ-LARC2 D6
C
Overlay
ACP
HSP70
DAPI
PfSPZ-WT D7
PfSPZ-LARC2 D7

Figure EV4. PfLARC2 LS display severe defects in late LS differentiation.

(**A**) The schematic depicts the experimental design. To evaluate LS development of PfLARC2 in FRG NOD huHep mice, three million aseptic, cryopreserved sporozoites of either PfSPZ-WT or PfSPZ-LARC2 were injected intravenously into two FRG NOD huHep mice per group, respectively. Livers were harvested on days 6 and 7 post infection, fixed and liver tissue sections used for IFA analysis. LS development of PfSPZ-LARC2 was compared to PfSPZ-WT using antibodies against (**B**), the PPM and cytomere marker, CSP and the PVM marker, Exp1 on day six liver sections, (**C**) parasite mitochondrial protein, heat shock protein 70 (HSP70, green), apicoplast protein, ACP (red) on day 7 liver sections. DNA is stained with DAPI. In (**B**) and (**C**), scale bar is 20 μm. Defective cytomeres were evident on day six PfSPZ-LARC2 LS compared to PfSPZ-WT, where CSP staining was localized to invaginating PPM. PfSPZ-LARC2 LS also displayed incomplete organellar and DNA segregation.

 © The Author(s)
